# Maternal gut microbiota‐derived daidzein prevents osteoporosis in female offspring following prenatal prednisone exposure

**DOI:** 10.1002/imt2.70037

**Published:** 2025-04-28

**Authors:** Chi Ma, Hangyuan He, Kunpeng Wang, Juanjuan Guo, Liang Liu, Yuting Chen, Bin Li, Hao Xiao, Xufeng Li, Xiaoqian Lu, Tingting Wang, Yinxian Wen, Hui Wang, Liaobin Chen

**Affiliations:** ^1^ Department of Orthopedic Surgery Division of Joint Surgery and Sports Medicine, Zhongnan Hospital of Wuhan University Wuhan China; ^2^ Department of Pharmacology Basic Medical School of Wuhan University Wuhan China; ^3^ Hubei Provincial Key Laboratory of Developmentally Originated Diseases Wuhan China; ^4^ Department of Obstetrics and Gynaecology Wuhan Hospital of Traditional Chinese Medicine Wuhan China

**Keywords:** daidzein, DOHaD, maternal gut microbiota, osteoporosis, prenatal prednisone exposure

## Abstract

Prenatal exposure to glucocorticoids is linked to long‐term health risks in offspring, but the role of maternal gut microbiota in mediating these effects remains unclear. Here, we demonstrate that prenatal prednisone therapy (PPT) in humans and prenatal prednisone exposure (PPE) in rats result in sex‐specific long bone dysplasia in offspring, including reduced peak bone mass (PBM) and heightened osteoporosis risk in female offspring. Multi‐omics profiling and fecal microbiota transplantation show that PPE alters maternal gut microbiota composition and depletes the microbial metabolite daidzein (DAI). DAI deficiency suppresses *Hoxd12* expression, impairs osteogenesis, and leads to PBM decline in female offspring. In bone marrow‐derived mesenchymal stem cells from PPE female offspring, DAI promoted *Hoxd12* expression and osteogenic differentiation. Notably, DAI supplementation restored H3K9ac levels, enhanced *Hoxd12* expression, and promoted osteogenic differentiation through the ERβ/KAT6A pathway. Furthermore, maternal DAI supplementation during pregnancy prevented osteoporosis susceptibility in PPE female offspring and alleviated functional abnormalities in multiple organs, including the liver, hippocampus, ovary, and adrenal gland. In conclusion, PPE induces multiorgan dysplasia and increases disease predisposition (e.g., osteoporosis) in female offspring by disrupting maternal gut microbiota and depleting DAI. Maternal DAI supplementation provides a promising preventive strategy to counteract these adverse outcomes.

## Highlights

- Prenatal prednisone therapy (PPT)/prenatal prednisone exposure (PPE) induces long bone dysplasia, reduced peak bone mass (PBM), and increased osteoporosis susceptibility in female offspring.
- Maternal gut microbiota metabolite daidzein (DAI) mediates PPE‐induced osteoporosis susceptibility in female adult offspring.
- DAI mediates the inhibition of osteoblast differentiation and increased susceptibility to osteoporosis in PPE offspring via ERβ/KAT6A‐mediated epigenetic regulation of *Hoxd12* expression.
- DAI emerges as a target for the early prevention and treatment of PPE‐induced multiorgan functional changes and related disease susceptibility.

## Graphical abstract

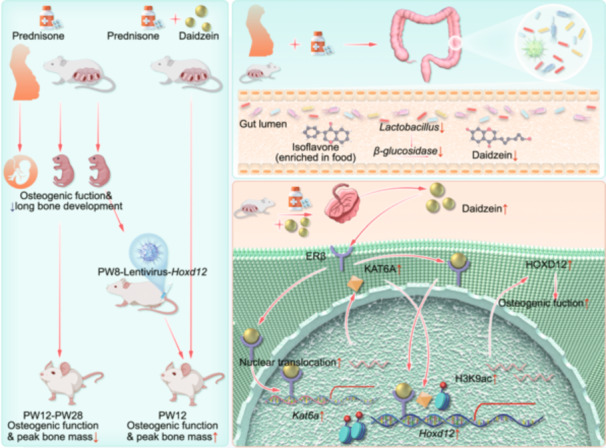

## INTRODUCTION

The “Developmental Origins of Health and Disease” (DOHaD) theory underscores the long‐term impacts of early‐life adverse conditions on offspring health and disease susceptibility. The gut microbiota, often referred to as the “second largest human genome” [1], plays a pivotal role in nutrient absorption, immune system regulation, and maintaining internal homeostasis. Growing evidence suggests that maternal gut microbiota during pregnancy influences the development of multiple organ systems in offspring, including the gastrointestinal, cardiovascular, immune, and nervous systems [2, 3, 4, 5, 6, 7]. Recent studies have demonstrated that adverse pregnancy conditions, such as drug exposure and high‐fat diets, can alter maternal gut microbiota, thereby increasing the risk of diseases like asthma, allergies, and obesity in offspring [2, 8, 9, 10]. Furthermore, Kimura et al. highlighted that offspring from germ‐free pregnant mice exhibit a significantly higher risk of developing metabolic syndrome in adulthood [11]. These findings suggest that maternal gut microbiota plays a pivotal role in offspring development, with adverse pregnancy conditions exacerbating the risk of multiple diseases through microbial alterations.

Postnatal bone mass is closely linked to adverse pregnancy conditions, such as prenatal perfluoroalkyl exposure [12]. Our previous research has shown that prenatal exposure to exogenous substances, including caffeine, nicotine, ethanol, and dexamethasone, results in low peak bone mass (PBM) and increased osteoporosis (OP) susceptibility in adulthood [13, 14]. A strong connection also exists between maternal gut microbiota and bone mass. Notably, when germ‐free pregnant C57BL/6 mice (a low‐bone‐mass strain) were transplanted with gut microbiota from pregnant C3H/HeN mice (a high‐bone‐mass strain), their offspring developed a high‐bone‐mass phenotype, and vice versa [7]. These findings emphasize the critical role of maternal gut microbiota in shaping offspring skeletal development and suggest that manipulating the gut microbiota could potentially optimize PBM. Prednisone, a widely used synthetic glucocorticoid, is commonly administered during pregnancy to manage rheumatic diseases, such as systemic lupus erythematosus, to enhance embryo implantation, and to prevent miscarriage [15]. The importance of using the lowest effective dose of prednisone during pregnancy is emphasized in many rheumatic conditions [16, 17]. Proper use of prednisone is essential to control disease activity in pregnant rheumatic patients while maintaining an optimal maternal environment for fetal development. It can be administered throughout pregnancy, including all trimesters. However, prenatal prednisone therapy (PPT) can increase the risks of placental insufficiency, premature birth, low birth weight, and cleft lip and/or palate [18, 19]. In animal models, prenatal prednisone exposure (PPE) has been shown to cause fetal ovarian developmental toxicity, articular cartilage impairments, and predisposition to osteoarthritis [20, 21, 22]. These findings highlight the “double‐edged sword” nature of prednisone use during pregnancy: while it is effective in managing maternal autoimmunity, it also carries developmental toxicity risks.

Prednisone has been shown to disrupt gut microbiota in rats, decreasing the relative abundance of genera such as *Eisenbergiella* and *Alistipes*, and reducing short‐chain fatty acid metabolites [23]. This raises the several questions of whether PPT or PPE affects offspring's long bone development and susceptibility to OP through alterations in maternal gut microbiota? What are the underlying mechanisms? How can OP susceptibility be prevented? To comprehensively assess the impact of PPT/PPE on maternal gut microbiota composition, offspring long bone development, and OP risk, clinical data and specimens, including fetal femoral development markers and maternal fecal samples, were collected from individuals with a history of prednisone use during pregnancy. Meanwhile, a PPE rat model was established by administering a clinical dose (0.25 mg/kg·d) of prednisone to Wistar rats throughout pregnancy. In this study, we established a novel and comprehensive study by examining the tripartite relationship between maternal gut microbiota dynamics, epigenetic regulatory mechanisms, and osteogenic differentiation processes in offspring. At the same time, we combined 16S rRNA sequencing with metabolomic profiling and transcriptomic analysis, complemented by systematic in vivo and in vitro validation experiments, to explore the mechanisms by which PPE‐induced offspring OP susceptibility. Further by fusing clinical case‐control data with preclinical models and molecular‐level analysis to create a chain of evidence supporting the scientific issues raised.

## RESULTS

### Alterations in maternal gut microbiota composition, offspring long bone dysplasia, and decreased PBM induced by PPE/PPT with sex‐specific differences

**FIGURE 1:**
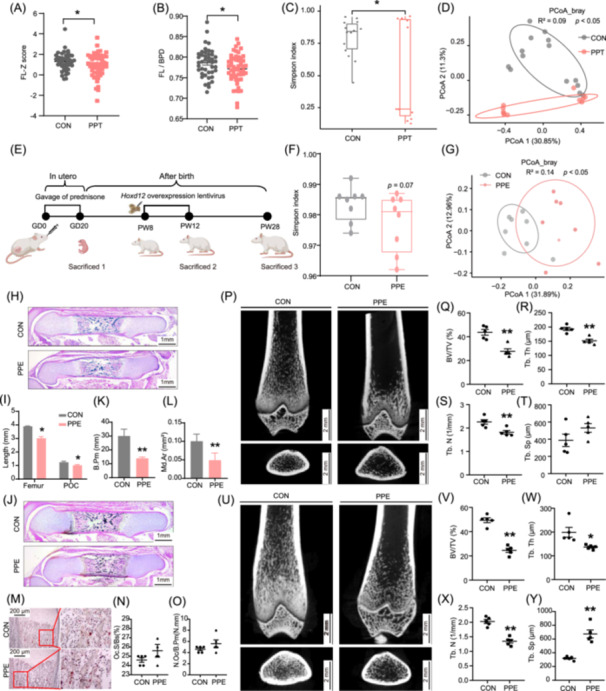
Effects of PPT/PPE on female long bone development and maternal gut microbiome. (A, B) Fetal FL‐Z score and FL/BPD ratio in human female fetus. (C) Boxplot of the Simpson index in human maternal gut microbiota. (D) PCoA plot analysis in human maternal gut microbiota. (E) Schematic diagram of PPE protocol. (F) Boxplot of the Simpson index in rat maternal gut microbiota. (G) PCoA plot analysis in rat maternal gut microbiota. (H, I) H&E staining of femur bone and quantitative analysis of the total femur and POC length, scale bar = 1 mm. (J–L) Vonkossa staining of femur and quantitative analysis of B. Pm and Md. Ar, scale bar = 1 mm. (M–O) Representative images of TRAP staining and quantitative analysis of Oc.S/Bs and N.Oc/B.Pm in GD20 female offspring femur, scale bar = 200 μm. (P–T) Representative images and quantitative analysis of micro‐CT for PW12 female offspring femur, scale bar = 2 mm. (U–Y) Representative images and quantitative analysis of micro‐CT for PW28 female offspring femur, scale bar = 2 mm. Data in (C, F) are presented as the median ± interquartile range, and data in other figures are presented as the Mean ± standard error of the mean (SEM), *n* = 111 in CON and *n* = 105 in PPT for case‐control study, *n* = 14 in CON and *n* = 11 in PPT for 16s rRNA sequencing of human maternal gut microbiota, *n* = 8 for 16s rRNA sequencing of rat maternal gut microbiota, *n* = 3 for H&E Staining and Vonkossa staining, *n* = 5 for micro‐CT. *p < 0.05, **p < 0.01 versus corresponding control. BPD, biparietal diameter; B. Pm, bone trabecula perimeter; BV/TV, bone volume/tissue volume; CT, computed tomography; CON, control; FL, femur length; GD, gestational day; H&E, hematoxylin and eosin; Md. Ar, mineralized area; N.Oc/B.Pm, osteoclast number per bone perimeter; Oc.S/Bs, osteoclast surface per bone surface; PPT, prenatal prednisone therapy; PPE, prenatal prednisone exposure; PCoA, principal co‐ordinates analysis; POC, primary ossification center; PW, postnatal week; TRAP, tartrate‐resistant acid phosphatase; Tb. N, trabecular number; Tb. Th, trabecular thickness; Tb. Sp, trabecular separation.

To elucidate the effects of PPT on pregnancy outcomes, as well as intrauterine fetal and bone development, a retrospective case‐control study was conducted, compiling a comprehensive data set of 223 maternal‐newborn cases (Table S1) for a rigorous intergroup analysis. The results revealed 28 preterm births in the PPT group, compared to only 1 in the control group. After excluding preterm births, the birth weights of both female and male neonates in the PPT group were lower than those in the control group. Analysis of late‐pregnancy B‐mode ultrasound results, following the methodology outlined in the literature [24, 25, 26], showed a significant reduced Z‐scores for femur length (FL‐Z) and the femur length (FL)/biparietal diameter (BPD) ratio in female fetuses in the PPT group (Figure 1A,B), while no significant changes were observed in male fetuses (Table S1). These results indicate that PPT is associated with an increased risk of preterm birth, low birth weight, and impaired long‐bone development in female fetuses. Maternal fecal samples collected during the second and third trimesters from another cohort of pregnant women (Table S2) were analyzed via 16S rRNA sequencing. Compared to the control group, the PPT group exhibited reduced α‐diversity and altered β‐diversity in their gut microbiota (Figure 1C,D). At the phylum level, there was a reduction in the relative abundance of beneficial bacteria such as o_*Lactobacillales* and g_*Akkemansia*, as determined by linear discriminant analysis effect size (LEfSe) (Figure S1A,B). Canonical correlation analysis (CCA) indicated that prednisone was the most significant factor contributing to the observed microbiota changes (32.8%) (Figure S1C). Collectively, these results suggest that maternal gut microbiota composition was altered in the PPT group, correlating with impaired long‐bone development in female fetuses.

To further validate these clinical findings and assess their long‐term effects, an animal study was performed using a clinical PPT regimen, where pregnant Wistar rats received 0.25 mg/kg·d of prednisone via oral gavage from gestational day (GD) 0 to 20 (Figure 1E). This model confirmed the clinical results and allowed investigation of the long‐term effects on PBM in adult offspring. While no significant changes were observed in the α‐diversity of the gut microbiota in the PPE group, β‐diversity was significantly altered (Figure 1F,G). A reduction in the relative abundance of beneficial bacteria such as s_*Lactobacillus_intestinalis* and s_*Clostridium_butyricum* was also noted (Figure S1D,E). The PPE group exhibited a significant reduction in FL, primary ossification center (POC) length, trabecular perimeter, and mineralized area in female fetal rats without altered osteoclastic activity (Figure 1H–O), with a continued decrease in PBM in adulthood at both PW12 and PW28 (Figure 1P–Y). Further analysis revealed that female offspring in the PPE group showed significantly reduced mRNA expression of osteogenic marker genes (*Runx2*, *Alp*, *Col1a1*, *Ocn*) and decreased RUNX2 protein expression in bone tissue, both pre‐ and post‐natally (Figure S2A–G). In contrast, bone development and PBM in male offspring showed no significant changes (Figure S2H–V). These results indicate that PPE‐induced alterations in maternal gut microbiota composition inhibit osteoblast differentiation, impair long bone development, and reduce PBM in female offspring, without affecting male offspring.

### Metabolite daidzein (DAI) of maternal microbiota is an intervention target for osteogenic function and PBM reduction in female offspring rats induced by PPE

**FIGURE 2:**
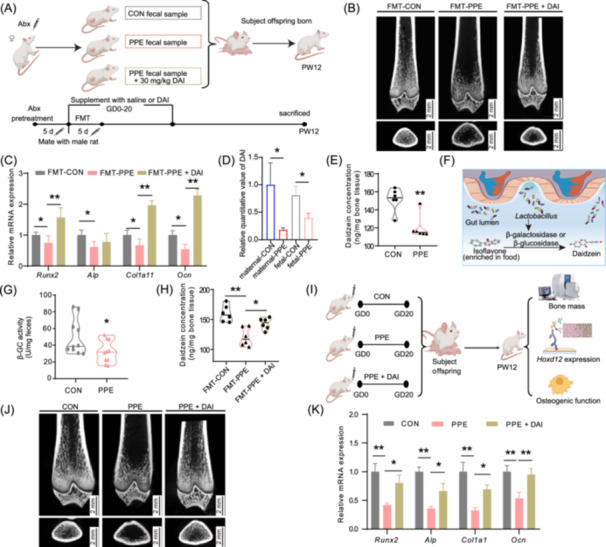
Involvement of maternal gut microbiome and DAI in downregulation of osteogenic function and low PBM in female offspring induced by PPE. (A) Schematic of the maternal FMT experiments. (B) PBM of PW12 offspring by micro‐CT, scale bar = 2 mm. (C) Osteogenesis differentiation‐related marker genes in PW12 offspring. (D) Relative quantitative value of DAI from metabolic profile. (E) DAI concentration in rat fetal bone shown as violin plots. (F) Liberation of isoflavone, which was enriched in food through β‐GC/β‐GAL. (G) β‐GC activity in rat maternal feces shown as violin plots. (H) DAI concentration in rat fetal bone is shown as violin plots. (I) Schematic of maternal DAI supplementation. (J) Representative images of micro‐CT for female offspring PBM, scale bar = 2 mm. (K) Relative expression of osteogenesis differentiation‐related marker genes. Data in (D, E, G, and H) are presented as the median ± interquartile range, and data in other figures are presented as the Mean ± SEM, *n* = 5 for micro‐CT, *n* = 8 for RT‐qPCR, *n* = 6 for untargeted metabolic profile of rat maternal and fetal serum. *n* = 30 in CON and *n* = 11 in PPT for LC‐MS of human maternal and fetal serum; *n* = 8 for LC‐MS of fetal bone, *n* = 10 for β‐GC activity assay. *p < 0.05, **p < 0.01 versus corresponding control. *Alp*, alkaline phosphatase; Abx, antibiotics; *Col1a1*, collagen type I Alpha 1; DAI, daidzein; *Hoxd12*, homeobox D12; PBM, peak bone mass; FMT, fecal microbiota transplant; β‐GC, β‐glucosidase; β‐GAL, β‐galactosidase; RT‐qPCR, real‐time quantitative polymerase‐chain‐reaction; FMT‐CON, pregnant rats transplanted with fecal microbiota from control maternal group; FMT‐PPE, pregnant rats transplanted with fecal microbiota from PPE maternal group; FMT‐PPE + DAI, pregnant rats transplanted with fecal microbiota from PPE maternal group and supplemented with DAI; *Runx2*, Runt‐related transcription factor 2; *Ocn*, Osteocalcin.

To explore the link between alterations in maternal gut microbiota composition and reduced osteogenic function and PBM in female offspring induced by PPE, a maternal fecal microbiota transplant (FMT) experiment was conducted (Figures 2A, S3A,B). The FMT‐PPE group exhibited sparser, thinner, and more widely spaced trabeculae, along with decreased BV/TV, Tb. Th, and Tb. N, and increased Tb. Sp compared to the FMT‐CON group. Additionally, osteogenic marker gene mRNA levels (*Runx2*, *Alp*, *Col1a1*, *Ocn*) and RUNX2 protein expression were significantly reduced (Figures 2B,C, S3C–H). These results suggest that alterations in maternal gut microbiota composition contribute to the inhibition of osteoblast differentiation, long bone dysplasia, and reduced PBM in female offspring rats exposed to PPE.

To further investigate how PPE‐induced changes in maternal gut microbiota affect osteoblast differentiation in female offspring, nontargeted metabolic profiling of maternal and fetal serum was performed (Figure S4A–F). The results revealed that DAI levels were significantly reduced in both maternal and fetal serum in the PPE group (Figure 2D), with similar reductions in DAI levels observed in the long bone tissue of female fetuses (Figure 2E). Further analysis showed a positive correlation between the relative abundance of *Lactobacillus* in maternal gut microbiota and DAI levels in fetal bone tissue (Figure S4G). Functional predictions based on PICRUSt indicated that PPE altered metabolic pathways of *Lactobacillus/Bifidobacterium*, while β‐glucosidase (β‐GC) activity in maternal gut microbiota was significantly reduced in the PPE group, with no significant changes in β‐galactosidase (β‐GAL) activity in PPE group (Figures 2F,G, S4H,I). These results suggest that the reduction in DAI levels in maternal/fetal serum and tissue following PPE may be linked to decreased β‐GC activity in the maternal gut microbiota. Moreover, supplementation with DAI in pregnant FMT‐PPE or PPE rats significantly reversed the reduction in DAI levels, osteoblast differentiation, and PBM in the long bone tissue of adult female offspring (Figures 2B,C,H–K, S3C–H, S5). These results support the hypothesis that DAI, a microbiome‐derived metabolite, is a potential intervention target for preventing the inhibition of osteoblast differentiation, long bone development, and PBM reduction in female offspring induced by PPE.

### *Hoxd12* is a key effector of DAI‐dependent osteogenesis impairment in female offspring exposed to prenatal prednisone

**FIGURE 3:**
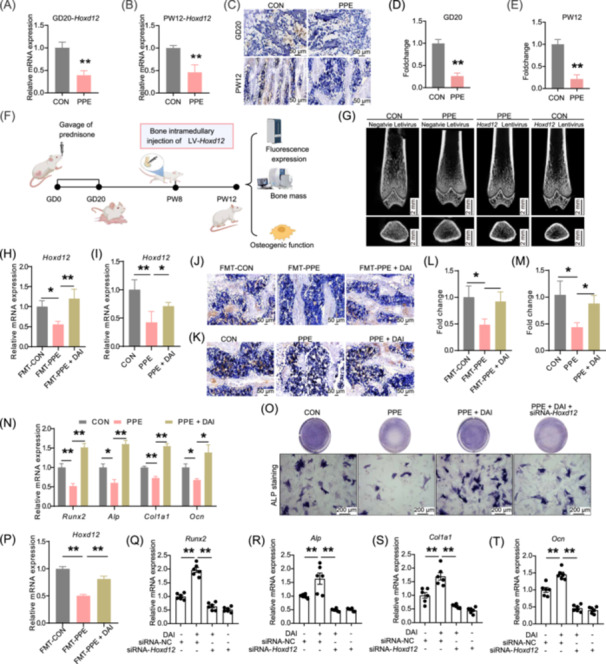
*Hoxd12* mediated the low osteogenesis function and PBM in female offspring rats induced by PPE. (A, B) Relative mRNA expression of *Hoxd12* at GD20 and PW12. (C) Representative immunohistochemical images of HOXD12, scale bar = 50 μm. (D, E) Quantitative analysis of HOXD12 in GD20 and PW12 female offspring. (F) Schematic of local injection eGFP‐labeled *Hoxd12* overexpressing lentivirus. (G) Representative images of micro‐CT. (H) *Hoxd12* mRNA expression in female offspring of FMT experiments. (I) *Hoxd12* mRNA expression in female offspring of maternal DAI supplementation experiments. (J) Representative immunohistochemical images of HOXD12 in female offspring of FMT experiments, scale bar = 50 μm. (K) Representative immunohistochemical images of HOXD12 in female offspring of maternal DAI supplementation experiments, scale bar = 50 μm. (L, M) Quantitative analysis of HOXD12 in PW12 female offspring. (N) Osteogenesis differentiation‐related marker genes in CON‐BMSCs and PPE‐BMSCs. (O) ALP staining of CON‐BMSCs and PPE‐BMSCs. (P) *Hoxd12* expression in CON‐BMSCs and PPE‐BMSCs. (Q–T) The effect of siRNA‐*Hoxd12* on expression of osteogenesis‐related marker genes. Mean ± SEM, *n* = 3 for RNA‐seq, *n* = 8 for RT‐qPCR, *n* = 3 for immunohistochemistry staining, *n* = 5 for micro‐CT, *n* = 3 for ALP staining. *p < 0.05, **p < 0.01 versus corresponding control. CON‐BMSCs, primary bone marrow mesenchymal stem cells in femur offspring from the control group; eGFP, enhanced green fluorescent protein; NC, negative control; PPE‐BMSCs, primary bone marrow mesenchymal stem cells in femur offspring from prenatal prednisone exposure group; siRNA, small interfering RNA.

Adverse prenatal environments can program the expression of critical genes in fetal bone tissue, leading to long‐term skeletal health issues such as OP [27]. Transcriptome sequencing of long bone tissue from female fetuses revealed that *Hoxd12*, a homeobox transcription factor gene involved in skeletal system development, was one of the most significantly downregulated genes in the PPE group (Figure S6A,B). RT‐qPCR and immunohistochemistry (IHC) further confirmed that *Hoxd12* expression remained persistently reduced in the bone tissue of PPE female offspring, both prenatally and postnatally (Figure 3A–E). In contrast, overexpression of *Hoxd12* in postnatal bone tissue significantly reversed the PPE‐induced inhibition of osteoblast differentiation and the reduction of PBM in female offspring (Figures 3F,G, S6C–J). Additionally, supplementation with DAI in pregnant rats from both FMT‐PPE and PPE groups significantly restored the reduced *Hoxd12* mRNA and protein expression in bone tissue (Figure 3H–M). These results suggest that *Hoxd12* mediates the effects of maternal microbiome‐derived DAI on osteoblast differentiation and PBM. Based on the physiological blood concentration of DAI [28], the effect of DAI on *Hoxd12* expression and osteoblast differentiation was investigated in primary bone marrow mesenchymal stem cells (BMSCs) derived from the long bones of offspring in both the control and PPE groups. Compared to CON‐BMSCs, PPE‐BMSCs showed inhibited osteoblast differentiation and reduced *Hoxd12* expression. Supplementing DAI significantly reversed these effects in PPE‐BMSCs. Moreover, silencing *Hoxd12* in PPE‐BMSCs counteracted the enhanced osteoblast differentiation induced by DAI supplementation (Figures 3N–T, S7). These results collectively indicate that *Hoxd12* mediates the enhancement of osteoblast differentiation induced by DAI in female offspring rats exposed to PPE.

### ERβ/KAT6A signaling mediates DAI‐induced epigenetic activation of *Hoxd12*

**FIGURE 4:**
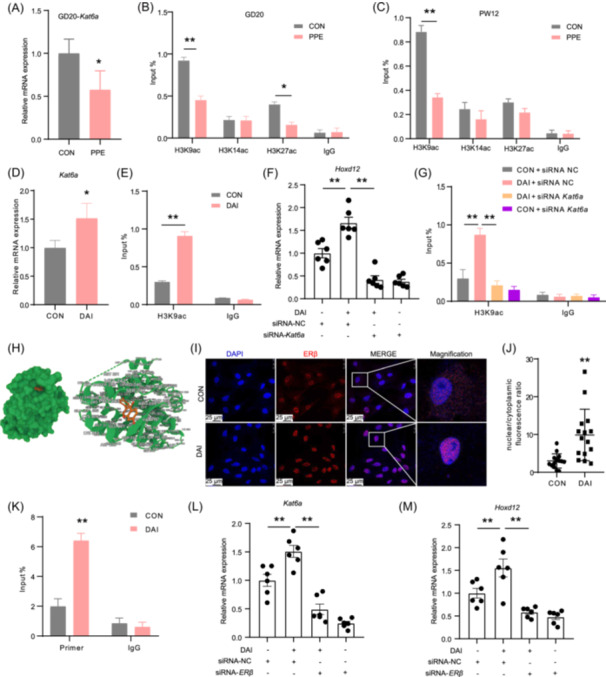
Upregulated *Hoxd12* in PPE‐BMSCs by daidzein (DAI) through ERβ/KAT6A. (A) *Kat6a* expression in female fetal offspring. (B, C) H3K9/14/27ac level in *Hoxd12* promoter region at GD20 and PW12. (D) *Kat6a* expression in BMSCs treated with or without DAI. (E) H3K9ac level in *Hoxd12* promoter region in BMSCs treated with or without DAI. (F) The effect of siRNA‐*Kat6a* on *Hoxd12* expression. (G) The effect of siRNA‐*Kat6a* on the H3K9ac level in *Hoxd12* promoter region. (H) Visualization of docking between DAI (red) and ERβ (green). (I, J) The location and nuclear/cytoplasmic fluorescence ratio of ERβ, scale bar = 25 μm. (K) Occupancy of ERβ upon DAI treatment at the *Hoxd12* promoter in PPE‐BMSCs. (L) The effect of siRNA‐*Erβ* on *Kat6a* expression. (M) The effect of siRNA‐*Erβ* on *Hoxd12* expression. Mean ± SEM, *n* = 3 for immunofluorescence staining, *n* = 6 for RT‐qPCR and ChIP‐PCR. *p < 0.05, **p < 0.01 versus corresponding control. ERβ, estrogen receptor β; *Kat6a*, lysine acetyltransferase 6A; H3K9/14/27ac, histone 3 lysine 9/14/27 acetylation.

Adverse prenatal environments induce programmed changes in multiple organ development through epigenetic modifications [29]. To explore the epigenetic mechanisms by which DAI reverses the low expression of *Hoxd12* induced by PPE, this study examined epigenetic databases (Cistrome Data Browser) and identified that *Hoxd12* is most commonly associated with histone modifications (Figure S8A). Transcriptome sequencing of fetal long bone tissue revealed that in the PPE group, the histone acetyltransferase *Kat6a* exhibited the most significant changes, with RT‐qPCR confirming a decrease in its expression (Figures 4A, S8B). Additionally, ChIP‐PCR analysis showed reduced levels of histone 3 lysine 9 acetylation (H3K9ac) at the *Hoxd12* promoter region both at GD20 and PW12 in the PPE group (Figure 4B,C). In vitro, DAI was shown to enhance *Kat6a* expression and elevate *Hoxd12* H3K9ac levels and expression (Figure 4D,E). However, silencing *Kat6a* significantly reversed the DAI‐induced increases in both *Hoxd12* H3K9ac levels and expression (Figures 4F,G, S8C–E). These results suggest that KAT6A mediates the effects of DAI on *Hoxd12* H3K9ac levels and its expression.

Given that DAI can act through estrogen receptor β (ERβ) [30], the ERβ‐mediated regulatory mechanisms were further explored. Molecular docking studies revealed that DAI can directly bind to ERβ (Figure 4H), and predictions from the JASPAR database (https://jaspar.genereg.net) suggested the presence of ERβ binding sites in the *Hoxd12* promoter region (Figure S8F). In vitro, DAI was found to promote ERβ translocation into the nucleus and its binding to the *Hoxd12* promoter, although no significant effect was observed on the protein level of ERβ (Figures 4I–K, S8G). Silencing ERβ significantly reversed the DAI‐induced increases in *Kat6a* and *Hoxd12* expression (Figures 4L,M, S8H–J). In summary, the activation of ERβ into the nucleus mediates the increase in *Hoxd12* H3K9ac levels and expression by regulating *Kat6a* expression in response to DAI.

## DISCUSSION

Due to the presence of 11β‐hydroxysteroid dehydrogenase 2 in the placenta, which inactivates glucocorticoids, only 10%–20% of prednisone typically crosses the placenta [31]. Consequently, prednisone is often recommended as a first‐line treatment for pregnant women with autoimmune diseases, such as rheumatoid arthritis (RA). A recent study revealed that the susceptibility of adult female offspring to osteoporosis (OP) induced by PPE is linked to changes in the skeletal muscle mitochondrial autophagy/FNDC5 axis [32]. However, the potential role of maternal gut microbiota in influencing offspring bone development and OP susceptibility through PPT or PPE remains unexplored, as does the identification of effective prevention and treatment strategies. Our findings in this study demonstrate that (1) PPT/PPE induces long bone dysplasia, reduced PBM, and increased OP susceptibility in female offspring; (2) Maternal microbial metabolite DAI mediates PPE‐induced reduction in PBM and OP susceptibility in female adult offspring; (3) DAI mediates the inhibition of osteoblast differentiation and increased susceptibility to OP in PPE offspring through ERβ/KAT6A‐mediated epigenetic regulation of *Hoxd12* expression; (4) DAI emerges as an early target for the prevention and treatment of PPE‐induced multiorgan functional changes and related disease susceptibility.

### PPT/PPE‐induced long bone dysplasia, PBM reduction, and OP susceptibility increase in female offspring

Clinical use of prednisone during pregnancy typically spans the entire duration, with doses ranging from 1 to 60 mg/day [15, 33, 34, 35]. Guidelines emphasize the use of the lowest effective dose to control maternal disease activity [16, 17]. PBM, which represents the bone mass achieved at the time of bone maturation, is a key determinant in the onset and progression of OP. Epidemiological studies suggest that a 10% increase in PBM can delay OP onset by 13 years, significantly reducing the risk of fractures associated with OP. Notably, postmenopausal women have a higher incidence and greater severity of OP [36, 37]. In the current study, clinical assessments based on neonatal birth weights and late‐pregnancy B‐mode ultrasound indicated that PPT inhibited female fetal long bone development. Additionally, based on clinical practices and the rat‐to‐human dosing conversion factor (6.17), 0.25 mg/kg·d of prednisone was administered via oral gavage throughout pregnancy. This treatment resulted in impaired osteoblast differentiation, decreased osteogenic mineralization in the POC, and reduced femoral length in female (but not male) fetal rats, consistent with clinical observations. Furthermore, PBM in adult offspring rats from the PPE group was reduced in females (but not males), suggesting that PPT/PPE can induce long bone dysplasia, PBM reduction, and increased OP susceptibility in female offspring.

### Maternal microbial metabolite DAI mediated PPE‐induced PBM reduction and OP susceptibility in female adult offspring

The maternal gut microbiota, as a heritable environmental factor, plays a critical role in offspring bone development and PBM. Abdul et al. demonstrated that maternal gut microbiota can directly influence offspring PBM through FMT experiments utilizing pregnant rat models with high and low PBM [7]. In the present study, PPT/PPE exposure was found to alter the maternal gut microbiota composition, notably reducing the abundance of beneficial bacteria such as *Lactobacillus*. FMT experiments further confirmed that these microbiota changes mediated PPE‐induced inhibition of osteoblast differentiation and reduced PBM in female offspring rats. In this study, we also observed that PPT/PPE can lead to changes in the composition of maternal gut microbiota, but these changes are not entirely consistent between humans and rats. This phenomenon may be attributed to lots of factors. Studies have reported that in late pregnancy, the abundance of *Akkermansia*, *Bifidobacterium*, and Firmicutes significantly increases in humans [38]. In contrast, as gestational age increases, the gut microbiota of rats shows a decrease in Firmicutes and *Verrucomicrobia* [39]. This suggests that the maternal gut microbiota undergoes adaptive changes with increasing gestational age, and there are compositional differences between the gut microbiota of humans and mice during pregnancy. Besides, human lifestyle factors, such as diet, exercise, and stress, have a significant impact on the composition and function of the gut microbiota. In comparison, the experimental conditions for rats are relatively uniform, lacking the complexity found in human life.

Gut microbiota metabolites serve as a key intermediary between maternal microbiota and fetal development [2, 6, 11]. Metabolomics, closely linked to phenotypic changes, provides an efficient approach to understanding how adverse prenatal environments affect fetal development. In this study, significant alterations in the metabolic profiles of maternal and fetal serum were identified in both the PPE and PPT groups through nontargeted and targeted metabolomic analyses (Table S3), with a marked decrease in DAI levels. DAI, a free isoflavone, is capable of crossing the placenta to influence fetal development [40, 41, 42, 43, 44, 45, 46]. It is widely present in maternal and fetal tissues, including urine, blood, amniotic fluid, breast milk, and umbilical cord blood, with its levels regulated by the gut microbiota [40, 47, 48, 49]. A prospective cohort study involving 480 mother‐infant pairs found that frequent maternal consumption of soy products resulted in higher urinary concentrations of isoflavones, such as DAI [43]. The study also revealed that the frequency of soy consumption correlated positively with neonatal weight, arm and waist circumference, and skinfold thickness. The levels of DAI in tissues are primarily determined by the ability of β‐GC or β‐GAL enzymes in the gut microbiota (e.g., *Lactobacillus_intestinalis*) to hydrolyze bound isoflavones from food [48].

Through multi‐omics analysis, this study identified a positive correlation between the relative abundance of *Lactobacillus_intestinalis* in maternal gut microbiota and DAI levels in fetal bone tissue. PICRUSt analysis indicated that PPE altered the metabolic pathways. Enzymatic assays confirmed a significant reduction in β‐GC activity in the gut microbiota of the PPE group. Additionally, supplementation with DAI in maternal rats from both the FMT‐PPE and PPE groups significantly reversed PPE‐induced decreases in DAI levels, osteoblast differentiation inhibition, and PBM reduction in female offspring long bone tissue. In conclusion, the maternal microbiota‐derived metabolite DAI mediates the reduction in PBM and increased susceptibility to OP in offspring induced by PPE, positioning DAI as a potential target for interventions aimed at preventing and treating fetal‐originated OP, and elucidation of the maternal‐fetal gut microbiota axis reveals novel intergenerational regulatory pathways.

### DAI mediated the inhibited osteoblast differentiation and susceptibility to OP in PPE offspring through ERβ/KAT6A epigenetic regulation of *Hoxd12* expression

To elucidate the epigenetic mechanisms through which low fetal blood DAI levels affect long bone development in offspring, previous studies have shown that *Hoxd* gene knockdown inhibits *Runx2* expression and osteogenic function [50], while *Hoxd12* knockout mice exhibit impaired skeletal development and reduced bone mass [51]. In the current study, transcriptome sequencing revealed a reduction in *Hoxd12* mRNA expression in fetal rat long bone, and further validation confirmed that both *Hoxd12* mRNA and protein levels were consistently decreased in the long bone of PPE female offspring before and after birth. Moreover, local overexpression of *Hoxd12* in the bone at PW8 reversed the inhibition of osteoblast differentiation and the reduction in PBM observed in PPE female offspring. Additionally, maternal DAI supplementation during pregnancy, or DAI supplementation to primary BMSCs, reversed the PPE‐induced reductions in *Hoxd12* expression, osteoblast differentiation, and PBM in the offspring. These findings collectively suggest that the low‐expression programming alterations of *Hoxd12*, both pre‐ and post‐natally, mediates PPE‐induced long bone dysplasia and PBM reduction in female offspring. Therefore, *Hoxd12* emerges as a key postnatal target for interventions aimed at preventing fetal‐originated OP susceptibility.

DAI, a phytoestrogen, acts as a selective ER agonist to modulate downstream gene expression. ERs exist in two subtypes, ERα and ERβ. ERα is predominantly expressed in cortical bone, while ERβ is more prevalent in cancellous bone and plays a more significant role in BMSC osteoblast differentiation [52]. Thus, this study focuses on ERβ. Adverse prenatal environments are known to increase offspring disease susceptibility through epigenetic modifications [29]. According to the Cistrome Data Browser, histone modification is the most common epigenetic modification associated with *Hoxd12*. Screening and validation via bone transcriptome sequencing revealed decreased expression of the histone acetyltransferase *Kat6a*, while ChIP‐PCR confirmed reduced H3K9ac levels at the *Hoxd12* promoter region in PPE female offspring before and after birth. *In vitro*, DAI was shown to increase *Kat6a* expression and *Hoxd12* H3K9ac levels, as well as *Hoxd12* expression. Silencing *Kat6a* reversed these effects. Additionally, DAI activated ERβ, promoting its binding to the *Hoxd12* promoter region. Silencing ERβ significantly reversed the DAI‐induced increases in *Kat6a* and *Hoxd12* expression.

**FIGURE 5:**
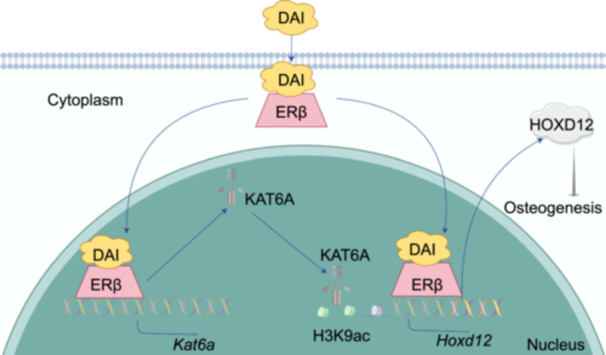
Schematic summary of daidzein (DAI) epigenetically regulates *Hoxd12* through ERβ/KAT6A to promote osteogenic differentiation in PPE‐BMSCs. DAI promotes *Hoxd12* transcription by activating ERβ, which translocates to the nucleus and binds to the *Hoxd12* promoter region, and DAI upregulates *Kat6a* expression through ERβ activation, leading to increased *Hoxd12* H3K9ac level and expression. The figure was created by Figdraw.

Taken together, DAI promotes *Hoxd12* transcription by activating ERβ, which translocates to the nucleus and binds to the *Hoxd12* promoter region, and DAI upregulates *Kat6a* expression through ERβ activation, leading to increased *Hoxd12* H3K9ac level and expression (Figure 5). Among them, the characterization of the ERβ/KAT6A signaling cascade promotes understanding of epigenetic controls in bone development.

Additionally, considering estrogen's role in intrauterine fetal development and its activating effect on ERβ in vivo, this study assessed fetal serum estrogen levels and found that PPE had no significant impact on the serum estrogen levels of female fetal rats. In vitro experiments further demonstrated that neither prednisone nor prednisolone significantly affected *Hoxd12* expression in BMSCs (Figure S9). These observations indicate that PPE‐induced reductions in *Hoxd12* expression and PBM in female offspring are not associated with fetal blood exposure to estrogen, prednisone, or prednisolone.

### DAI is an early target for the prevention and treatment of PPE‐induced multiorgan functional changes and susceptibility to related diseases

A recent review highlighted that drug exposure during pregnancy can lead to developmental toxicity in offspring, affecting multiple organs and increasing susceptibility to various diseases in adulthood. Different organs exhibit varying sensitivities to the same drug [53]. In the present study, PPE resulted in inhibited osteoblast differentiation and reduced PBM in female offspring, along with increased hepatic lipid synthesis, impaired hippocampal neurodevelopment, enhanced adrenal steroid synthesis, and ovarian follicular dysplasia (Figure S10A–G). These results suggest that PPE disrupts functional homeostasis across multiple organs, including the long bones, liver, hippocampus, adrenal glands, and ovaries in offspring. The serum level of DAI in female infants positively correlates with their birth weight (Figure S10H). Furthermore, maternal supplementation with DAI reversed PPE‐induced reductions in PBM and the functional alterations in multiple organs, including hepatic lipid synthesis, hippocampal neurodevelopment, adrenal steroid synthesis, and ovarian follicular development. These results indicate maternal DAI supplementation effectively promotes fetal development and prevents susceptibility to multiple diseases in offspring post‐birth. Therefore, DAI supplementation during pregnancy may serve as a common target to counteract PPE‐induced fetal‐originated diseases. The findings also suggest that for pregnant women with autoimmune diseases undergoing prednisone treatment, daily intake of isoflavone‐rich foods, such as soy products or tofu, could help prevent disturbances in organ function homeostasis in offspring [41, 54]. However, given that DAI is a phytoestrogen, there are legitimate concerns that it may act as an endocrine disruptor and affect the health homeostasis of offspring [30]. Therefore, when supplementing pregnant mothers with DAI, it is crucial to strictly control the dosage of DAI and dynamically monitor the levels of DAI in maternal blood to avoid adverse effects on the fetus due to excessive DAI supplementation.

### Limitations of this study

However, several limitations exist in the current study. First, the impact of PPT on fetal long bone development was assessed through a retrospective case‐control study, and the effect of PPT on postnatal bone mass accumulation in offspring was not investigated, due to the long latency of fetal‐originated diseases. Future research will employ multidisciplinary collaboration across obstetrics, nutrition, pediatrics, and orthopedics, combined with multicenter clinical cohort studies, to explore the relationship between PPT and postnatal bone mass accumulation in offspring. Second, while in vivo and in vitro experiments suggest that maternal supplementation with DAI is an effective strategy to prevent multiorgan homeostasis alterations in offspring, further clinical studies with larger sample sizes are necessary to confirm the safety and efficacy of this approach.

## CONCLUSION

This study provides the first evidence that PPT/PPE exposure reduces maternal microbial metabolite DAI levels by altering the composition of the maternal gut microbiota (e.g., *Lactobacillus*) and inhibiting β‐GC activity. The resultant decrease in DAI levels in fetal blood induces low‐functional programming of *Hoxd12*, leading to inhibited osteoblast differentiation, long bone dysplasia, and reduced PBM. In vitro, it was confirmed that DAI exerts its effects by activating ERβ, which upregulates KAT6A. This, in turn, increases the H3K9ac levels and expression of *Hoxd12*, promoting osteoblast differentiation. Moreover, in vivo experiments demonstrated that maternal DAI supplementation during pregnancy not only effectively prevented osteoblast differentiation inhibition, PBM reduction, and OP susceptibility in female PPE offspring but also mitigated dysfunction in other organs, including the liver, hippocampus, adrenal glands, and ovaries. The study establishes a link among the microbiota‐epigenetics‐bone development axis, providing novel target for OP prevention strategies. The innovative exploration of DAI as a therapeutic candidate has demonstrated its translational potential to prevent fetal‐originated OP. It is suggested that supplementation of gut microbial metabolites during pregnancy may serve as an effective strategy to prevent and mitigate fetal development alterations induced by prenatal glucocorticoid exposure, to emphasize the importance of maternal microenvironment optimization during pregnancy.

## METHODS

### Chemicals and reagents

Prednisone (LA21334) was obtained from Xianju Pharmaceutical Co., Ltd. DAI (CAS: 486‐66‐8, purity ≥ 98%, MW: 254.24) and was purchased from RHAWN®. Isoflurane (R510‐22‐10) was sourced from Baxter Healthcare Co. Taq Pro Universal SYBR qPCR Master Mix (Q712‐02) and HiScript III RT SuperMix for qPCR (R323‐01) was acquired from Vazyme Biotech Co., Ltd. Lipofectamine® 3000 Reagent (L3000015), TRIzol TM Reagent (15596026), α minimum essential medium (12571063), phosphate‐buffered saline (10010023), and fetal bovine serum (A5670402) were supplied by Thermo Fisher Scientific Co., Ltd. The Omni‐ECL™ Femto Light Chemiluminescence Kit (SQ101L) was purchased from Epizyme Biotech. The *Er*β siRNA, *Kat6a* siRNA, and *Hoxd12* siRNA were sourced from Tsingke Biotech Co., Ltd. All other chemicals used were of analytical grade and commercially available. The antibody against HOXD12 (Ab230697) was purchased from Abcam Co. The antibodies against RUNX2 (A11753), COL1A1 (A24112), and Erβ (A26153) were purchased from Abclonal. Antibodies for H3K9ac (No. A7255), H3K14ac (No. A7254), H3K27ac (No. A7253), rabbit control IgG (No. AC005), and glyceraldehyde‐3‐phosphate dehydrogenase (GAPDH, No. AC002) were also obtained from Abclonal.

### Clinical study

The study was approved by the Zhongnan Hospital Medical Ethics Committee of Wuhan University (NO. LYL[2023141K]). Data were extracted, including medication history, neonatal sex, birth weight, and prenatal ultrasound results. The inclusion criteria encompassed pregnant women aged 20–35 years, with singleton pregnancies who regularly attended antenatal check‐ups at the hospital's outpatient department and had access to late‐pregnancy B‐mode ultrasound results. Exclusion criteria included cases of multiple pregnancies, pregnancies complicated by diabetes, hypertension, prenatal diagnoses confirming fetal malformations or other diseases, and a history of glucocorticoid use during pregnancy, including dexamethasone, betamethasone, and hydrocortisone. A total of 223 pairs of maternal‐neonatal case data were collected (Table S1) and divided into the control and PPT groups based on prenatal prednisone medication usage. In the PPT group, pregnant women received oral prednisone at doses ranging from 2 to 10 mg/d. To account for gestational age as a confounding factor, the FL Z‐score and FL/BPD ratio were calculated, referencing established standards, to match fetal growth and development indices [25, 26].

For the collection of maternal feces, 25 participants were recruited from the Obstetrics Outpatient Departments at Zhongnan Hospital of Wuhan University and Wuhan Hospital of Traditional Chinese Medicine (Table S2). Inclusion and exclusion criteria mirrored those of the case‐control study, and informed consent was obtained through signed paper‐based consent forms. Maternal fecal samples were collected in sterile tubes containing a preservative solution (MGI Tech Co., Ltd; A0212) and transferred to a −80°C freezer within 24 h for subsequent 16S sequencing analysis.

For the acquisition of clinical blood samples, 41 pairs of maternal‐female infant samples were enrolled from the Obstetrics Department at Zhongnan Hospital of Wuhan University (Table S3). The grouping, inclusion, and exclusion criteria were similar to those of the case‐control study, and informed consent was obtained from the families via signed consent forms. Both maternal and female infant umbilical cord serum were collected and stored at −80°C for later quantification of DAI levels.

### Animals and treatment

Specific pathogen‐free Wistar rats (No. 110324210100788048) were purchased from SpePharm, with males weighing 280 ± 20 g and females weighing 240 ± 10 g. All animal procedures were approved by the Institutional Animal Care and Use Committee (IACUC) of Wuhan University (No. WP20210060) and conducted in accordance with the National Institutes of Health (NIH) Guidelines for the Care and Use of Laboratory Animals and Animal Research Reporting of In Vivo Experiments (ARRIVE) guidelines. The rats were maintained under standard conditions (temperature: 18°C–22°C, humidity: 40%–60%, 12‐h photoperiod). After a 1‐week acclimatization period, male and female rats were mated in a 2:1 ratio. On the following day, spermatozoa were detected in vaginal smears, marking GD0. In the PPE group, rats were gavaged with prednisone (0.25 mg/kg·d) from GD0 to GD20, while the control group received an equal volume of sodium carboxymethylcellulose solvent.

On GD20, some pregnant rats were anesthetized with 2% isoflurane and underwent cesarean section for fetal collection. Only pregnant rats with litters of 8–14 pups were included in the study. Fetal femurs from different litters were collected for analysis. The remaining rats, including those in the PPE and control groups, gave birth spontaneously. To ensure adequate nutrition for the lactating pups, the litter size was adjusted to 12 pups per litter (with an approximately equal male‐to‐female ratio) from PD1 to PW4. After weaning, two female and two male pups per litter were randomly selected and euthanized at PW12 and PW28, respectively. Additionally, one female pup from each litter was selected for lentiviral injection (10 μL, 2.91 × 10^8^ TU/mL) of either *Hoxd12* overexpression lentivirus or negative control lentivirus into the distal femur and proximal tibia at PW8 and PW9. The pups were euthanized 4 weeks after lentiviral injection, and their femurs were collected for further analysis. The experimental timeline is illustrated in Figure 1E.

### Faecal microbiota transplantation (FMT)

FMT experiments were conducted using pseudo‐sterile rats, which were prepared following previously described methods [55, 56]. Briefly, 6–8 week‐old specific pathogen‐free female Wistar rats were gavaged daily for five consecutive days with a broad‐spectrum antibiotic solution containing 100 mg/kg vancomycin, 200 mg/kg neomycin sulfate, 200 mg/kg metronidazole, and 200 mg/kg ampicillin. Considering prednisone's low lipophilicity and minimal intestinal retention properties [57], along with its half‐life of approximately 3–4 h, GD20 maternal intestinal contents were collected at 12 h after prednisone administration to ensure complete drug clearance. The administration volume was 0.1 mL/10 g body weight. Pre‐ and post‐antibiotic fecal samples were cultured on BHI agar plates under both anaerobic and aerobic conditions to verify the success of microbiota depletion. After completing the antibiotic regimen, pseudo‐sterile female rats were mated with male rats. FMT was performed from GD0‐5, as previously reported [7, 55]. Briefly, fresh fecal samples were collected from control and PPE pregnant rats on GD20 using a sterile fecal collector. The samples were resuspended in sterile PBS (7.5 mL/g feces) in a clean bench, vortexed for 5 min, and then centrifuged at 600 *g* for 3 min. The supernatant was collected, and pseudo‐sterile rats were gavaged daily for five consecutive days with the prepared supernatant.

### Bone histomorphometry

Following sacrifice, the femurs were fixed in 4% paraformaldehyde. For adult rats, femurs were decalcified in 10% EDTA for 12 days. Both fetal and adult femurs were embedded in paraffin, and 4 μm sections were prepared along the sagittal axis. Hematoxylin‐eosin (H&E), Von Kossa, and tartrate‐resistant acid phosphatase (TRAP) staining, staining were performed as described previously [58, 59], and bone histomorphometry analyses were conducted. Five random regions of each sample were imaged using an Olympus AH‐2 light microscope, and the results were analyzed with Image‐Pro Plus software (version 6.0).

### Immunohistochemical analysis

For immunohistochemistry, bone tissue samples were sequentially dehydrated, paraffin‐embedded, and sectioned at 4 μm. Sections were deparaffinized and subjected to antigen retrieval in a citric acid solution (pH 6.0). After washing three times in PBS, the sections were treated with 3% hydrogen peroxide at room temperature for 20 min, followed by blocking with 3% BSA for 1 h. Primary antibodies were incubated overnight at 4°C, followed by incubation with HRP‐labeled secondary antibodies for 1 h. The sections were then washed three times in PBS and incubated with freshly prepared DAB solution (Servicebio) for 5 min. Positive staining was indicated by brownish‐yellow color. After counterstaining with hematoxylin, five random regions of each sample were imaged by an Olympus AH‐2 light microscope (Olympus), and results were analyzed using Image‐Pro Plus software (version 6.0).

### Immunofluorescence staining

Antigen retrieval for tissue sections was performed using a citric acid antigen repair solution (pH 6.0). Cell samples were fixed with 4% paraformaldehyde at room temperature for 15 min. Both bone tissue and cell samples were permeabilized with 0.1% Triton X‐100 and blocked with 3% BSA for 60 min at room temperature. Following three washes with PBS, the samples were incubated overnight at 4°C with primary antibodies for HOXD12 (1:100), ERβ (1:100), and RUNX2 (1:100). The next day, the samples were incubated with CY3‐labeled fluorescent secondary antibody (1:200) for 1 h at room temperature after three PBS washes. Five random regions of each sample were imaged using a Leica TCS SP8 X White Light Laser Confocal Microscope or a Nikon H550S photo imaging system (Japan). The average optical density was quantified using Image‐Pro Plus software (version 6.0).

### Microcomputed tomography (micro‐CT)

Femoral samples were scanned using high‐resolution micro‐CT (Skyscan1276, Bruker Biospin) with the following parameters: source voltage 50 kV, source current 200 μA, image pixel size 20 μm, and an energy filter of 1.0. The region of interest was defined as 200 slices starting from the distal femoral epiphyseal growth plate. Bone morphology and microarchitecture were analyzed using CTAn software (version 1.18.4), with parameters including BV/TV, Tb. N, Tb. Th, and Tb. Sp.

### 16s rRNA microbiome sequencing

Genomic DNA was extracted from the samples using CTAB or SDS methods, and the purity and concentration were evaluated by 1% agarose gel electrophoresis. The V3‐V4 region of the 16S rRNA gene was amplified with the following primers: F: 5′‐CCTAYGGGRBGCASCAG‐3′ and R: 5′‐GGACTACNNGGGTATCTAAT‐3′. After quantification, PCR products were used to generate sequencing libraries with the NEBNext® Ultra™ II DNA Library Prep Kit (Cat No. E7645). The library was qualified and sequenced on the Illumina NovaSeq. 6000 platform by Novogene Biotechnology Co., Ltd.

### Untargeted metabolomics

A 400 μL aliquot of 80% aqueous methanol was added to 100 μL of serum samples in EP tubes. The mixture was vortexed and shaken, then incubated on ice for 5 min. Following incubation, the samples were centrifuged at 15,000 *g* for 20 min at 4°C. The supernatant was transferred to a new EP tube and diluted to 53% methanol with mass spectrometry‐grade water. After a second centrifugation at 15,000 *g* for 20 min at 4°C, the supernatant was collected and injected into an LC‐MS for analysis. UHPLC‐MS/MS was performed using a Vanquish UHPLC system (Thermo Fisher) coupled with an Orbitrap Q Exactive^TM^ HF mass spectrometer (Thermo Fisher). Seven quality control (QC) samples, prepared by mixing equal volumes of experimental samples, were analyzed during the experiment to equilibrate the chromatography‐mass spectrometry system, monitor instrument status, and ensure system stability. A blank sample was also analyzed to remove background ions. Serum samples were injected into a Hypersil Gold C18 column. For positive polarity mode, eluent A was 0.1% FA in water, and eluent B was methanol; for negative polarity mode, eluent A was 5 mM ammonium acetate (pH 9.0), and eluent B was methanol.

The raw data were processed using Compound Discoverer 3.1 (CD3.1; Thermo Fisher) to obtain qualitative and quantitative results for the metabolites. Data quality was monitored and controlled for accuracy and reliability. Statistical analyses, including principal component analysis and variable importance in the projection (VIP) from the OPLS‐DA model, were performed to assess metabolic differences among groups. Metabolites with VIP > 1 and fold changes > 2 were considered significantly altered.

### Liquid chromatography/tandem mass spectrometer (LC‐MS/MS)

The concentration of DAI was measured as previously described [60, 61]. Briefly, the pretreated sample was analyzed using an LC‐MS/MS system (TSQ Quantis, Thermo Scientific) under the following conditions: C18 column (4.6 mm, 150 mm, 5 μm), column temperature 30°C, and a mobile phase consisting of acetonitrile and 1% aqueous phosphoric acid (30:70) at a flow rate of 1 mL/min.

### RNA sequencing analysis

Fetal bone RNA from the control and PPE groups were extracted and sequenced on an Illumina NovaSeq platform by Novogene Biology Information Technology Co., Ltd. Differential expression analysis was conducted using the DESeq. 2R package (1.20.0), and genes with adjusted *p*‐values were considered significantly differentially expressed. The clusterProfiler R package was used for Gene Ontology (GO) and Kyoto Encyclopedia of Genes and Genomes enrichment analyses of differentially expressed genes.

### Primary BMSCs isolation, culture, and treatment

Primary BMSCs were isolated, cultured, and induced for osteogenic differentiation as previously described [14]. Briefly, rat femora and tibiae were dissected, with muscle tissue removed. After rinsing the bones three times with PBS, the epiphyses were removed, and the bone marrow cavity was flushed with α‐MEM medium. The resulting marrow suspension was collected and centrifuged at 1000 rpm for 3 min at room temperature to discard the supernatant. The pellet was washed twice more with PBS, then resuspended in α‐MEM medium containing 10% FBS and 1% penicillin/streptomycin. Cells were cultured at 37°C in a 5% CO_2_ incubator. For osteogenic differentiation, BMSCs were seeded in 12‐well plates and cultured until they reached 60%–80% confluence. Osteogenic induction medium (α‐MEM supplemented with 10% FBS, 1% penicillin/streptomycin, 10 nM dexamethasone, 50 μg/L Vitamin C, and 10 mM sodium β‐glycerophosphate) was added, and cells were incubated for 2 weeks. During osteogenic differentiation, BMSCs were treated with prednisone, prednisolone, or DAI.

### Small interfering RNA (siRNA) knockdown of *Kat6a*, *Hoxd12*, and *Erβ*

To knock down the expression of *Kat6a*, *Hoxd12*, and *Er*β, siRNA transfection was performed. BMSCs were plated in six‐well plates and cultured in α‐MEM medium until cell confluence reached 60%–80%. SiRNA targeting *Kat6a*, *Hoxd12*, and Erβ was transfected using Lipofectamine 3000 for 6 h. The silencing efficiency was confirmed by RT‐qPCR. The siRNAs were obtained from Beijing Tsingke Biotech Co., Ltd., and the target sequences were as follows: *Erβ*‐1: F: 5′‐CACGAATCAGTGTACCATA‐3′, *Erβ*‐2: F: 5′‐GTCCTGCTGTGATGAACTA‐3′, *Erβ*‐3: F: 5′‐CACCTTGAGTCCAGAGCAA‐3′; *Kat6a*‐1: F: 5′‐GCGCTATGCTAATCCAATA‐3′, *Kat6a*‐2: F: 5′‐CTTCCACACGAGAAAGATA‐3′, *Kat6a*‐3: F: 5′‐GGCGAATAGCACTTCCTAA‐3′; *Hoxd12*‐1: F: 5′‐GGTTCACTCGGCTCTCAAA‐3′, *Hoxd12*‐2: F: 5′‐GACCAGGTCAAGTTCTATA‐3′, *Hoxd12*‐3: F: 5′‐CTTCAAGGAAGACACCAAA‐3′.

### Alizarin red S (ARS) staining

To assess osteogenic differentiation, ARS staining was performed after 2–3 weeks of osteogenic induction. Briefly, cells were washed with PBS and fixed at room temperature for 20 min. After another PBS wash, cells were stained with ARS solution for 30 min at room temperature. The samples were washed with distilled water, observed, and photographed under a Nikon microscope (ECLIPSE TE2000‐E; Japan). Red mineralized nodules indicated positive staining.

### Alkaline phosphatase (ALP) staining

ALP staining was performed after 10 days of osteogenic induction using the BCIP/NBT ALP Color Development Kit (MA0197; Meilunbio). Cells were washed with PBS, fixed with 4% paraformaldehyde for 5–10 min, and washed three times with PBS. After PBS removal, 400 μL of ALP staining solution was added to each well and incubated at room temperature for 30 min in the dark. The samples were then washed with PBS and photographed under a Nikon microscope (ECLIPSE TE2000‐E; Japan). Positive staining appeared as dark blue to bluish purple.

### RNA isolation and quantitative RT‐qPCR

Total RNA from bone or BMSCs was isolated using the TRIzol reagent kit (Invitrogen) following the manufacturer's protocol. Briefly, 1 mL of TRIzol reagent and 200 μL of chloroform were added to the prepared samples, which were then shaken thoroughly and incubated on ice for 10 min. After centrifugation at 12,000 rpm for 15 min, the supernatant was collected, and 400 μL of isopropanol was added to precipitate the RNA. The mixture was incubated at room temperature for 10 min and then centrifuged again at 12,000 rpm for 10 min. The supernatant was discarded, and the RNA was washed twice with 1 mL of pre‐cooled 75% ethanol. RNA concentration and quality were determined using a NanoDrop One/OneC Microvolume UV–Vis Spectrophotometer (Thermo Scientific). For cDNA synthesis, 1 μg of total RNA from each sample was reverse‐transcribed using the HiScriptⅡ 1st Strand cDNA Synthesis Kit (R212‐01; VAZYME). RT‐qPCR was performed using the Taq Pro Universal SYBR qPCR Master Mix (Q712‐02, VAZYME) on a QuantStudio 6 Flex Real‐Time PCR System (Thermo Scientific). The PCR conditions were as follows: 95°C for 30 s, followed by 40 cycles of 95°C for 10 s, and 62°C for 30 s. The relative mRNA expression was normalized to GAPDH using the formula *Y* = 2^−^^Δ^ ^Δ^ ^Ct^. Primer sequences for RT‐qPCR are provided in Table S4.

### Western blot analysis

For protein extraction, cell samples were washed three times with cold PBS and lysed in RIPA buffer containing 1 mM PMSF (Beyotime). The samples were incubated on ice for 20 min, then centrifuged at 12,000 rpm for 10 min at 4°C to obtain the supernatant. Protein concentration was measured using the BCA Protein Assay Kit (ZJ101, Epizyme Biotech). Protein samples (30 μg) were subjected to 10% sodium dodecyl sulfates polyacrylamide gel electrophoresis and transferred onto a membrane. The membrane was blocked with Protein Free Rapid Blocking Buffer (PS108P, Epizyme Biotech) for 5 min at room temperature and incubated overnight at 4°C with primary antibodies against ERβ (1:1000) and GAPDH (1:2000). After washing with Tris‐Buffered Saline and Tween 20 (TBS‐T) solution, the membrane was incubated with an HRP‐linked secondary antibody (1:8000 dilution) for 1 h at room temperature. The bands were visualized using the Omni‐ECL™Pico Light Chemiluminescence Kit (SQ202, Epizyme Biotech), and band intensities were quantified using Image J software.

### Molecular docking analysis

The binding energy and interaction pattern between DAI and ERβ were assessed using Autodock Vina 1.2.2 software (http://autodock.scripps.edu/). The molecular structure of DAI (PubChem CID 5281708) was obtained from the PubChem Compound Database (https://pubchem.ncbi.nlm.nih.gov/), and the 3D coordinates of the ERβ protein (PDB: 1QKN, Resolution: 2.25 Å) were downloaded from the Protein Data Bank (http://www.rcsb.org/). To prepare the protein and ligand files for docking, both were converted to PDBQT format, all water molecules were removed, and polar hydrogen atoms were added. The docking grid box was centered to cover the structural domains of ERβ and to accommodate free molecular motion. The grid box was set to a 30 Å × 30 Å × 30 Å square with a grid point distance of 0.05 nm. Molecular docking was performed using Autodock Vina 1.2.2, and the interaction patterns and binding energy were visualized.

### Chromatin immunoprecipitation‐qPCR (ChIP‐PCR) assay

ChIP‐PCR was performed as previously described [59]. Bone tissue and BMSC samples were cross‐linked with 1% formaldehyde for 10 min, followed by the addition of 0.125 M glycine to terminate cross‐linking for 5 min at room temperature. After centrifugation at 3000 rpm for 2 min, the supernatant was discarded. The cell lysates were sonicated (30% power, 2 s on, 1 s off, for 5 min) to yield approximately 200 bp DNA fragments. Following centrifugation at 12,000 rpm for 10 min at 4°C, the supernatant was transferred to a new tube. A 10 μL aliquot of the supernatant was reserved as “Input” and stored at 4°C. The remaining 100 μL was incubated overnight at 4°C with Protein G‐blocked beads and antibodies (IgG, H3K4ac, H3K9ac, H3K27ac) on a rotary shaker (20–30 rpm). Beads were then collected and eluted from the immunoprecipitation complexes using elution buffer containing 200 μg/mL proteinase K. After elution, samples were subjected to overnight incubation at 65°C in a water bath to reverse cross‐linking. The isolated DNA was purified using the PCR Clean Kit (Beyotime Biotechnology, Shanghai, China) and amplified via RT‐qPCR with *Hoxd12* primers. Primer sequences were as follows: F: 5′‐GATGCACCCTTAGGCCTGTA‐3′, R: 5′‐GCATCCTCTGGCATGAATTT‐3′. The rat primers for assessing the binding of *ERβ* to the *Hoxd12* promoter region (nt‐867/‐853) were: F: 5′‐TGGTCGCCATAACTCACACA‐3′, R: 5′‐AGAGACTGTGTGATGGGAGC‐3′. Results were calculated using the formula “Input % = 100 × 2^(Ct input – Ct IP)^.”

### Statistical analysis

Data were analyzed using SPSS 20 and GraphPad Prism 8.0 software. Data are presented as the Mean ± standard error of the mean unless otherwise noted [13]. If the data were normally distributed with homogeneity of variance, comparisons between two groups were made using Student's *t*‐test, and one‐way analysis of variance was used for multiple comparisons with Dunnett or Tukey post hoc tests. For quantitative data with no homogeneity of variance or non‐parametric distribution and qualitative data, the Kruskal–Wallis *H* test was used before pairwise comparison with the *Nemenyi* test. A *p*‐value < 0.05 (two‐tailed) was considered statistically significant.

## AUTHOR CONTRIBUTIONS

**Chi Ma**: Writing—original draft; conceptualization; investigation; methodology; project administration. **Hangyuan He**: Conceptualization; methodology; software. **Kunpeng Wang**: Methodology; software; data curation. **Juanjuan Guo**: Validation; data curation; formal analysis. **Liang Liu**: Formal analysis; project administration. **Yuting Chen**: Formal analysis; project administration. **Bin Li**: Project administration; formal analysis; data curation. **Hao Xiao**: Data curation; formal analysis; project administration. **Xufeng Li**: Project administration; formal analysis; data curation. **Xiaoqian Lu**: Project administration; formal analysis; software. **Tingting Wang**: Project administration; formal analysis; software; methodology; data curation; visualization. **Yinxian Wen**: Project administration; formal analysis; data curation; software; visualization. **Hui Wang**: Writing—review and editing; funding acquisition; resources; supervision. **Liaobin Chen**: Writing—review and editing; funding acquisition; resources.

## CONFLICT OF INTEREST STATEMENT

The authors declare no conflicts of interest.

## ETHICS STATEMENT

The ethics application of the clinical study (No. LYL[2023141K]) was approved by the Zhongnan Hospital Medical Ethics Committee of Wuhan University. All animal procedures were approved by the Institutional Animal Care and Use Committee (IACUC) of Wuhan University (No. WP20210060) and conducted in accordance with the NIH Guidelines for the Care and Use of Laboratory Animals and ARRIVE guidelines.

## Supporting information


**Figure S1.** Effects of PPT/PPE on maternal gut microbiome, male offspring long bone development, and PBM in male offspring.
**Figure S2.** Effects of PPE on osteogenesis function in female offspring.
**Figure S3.** Involvement of maternal gut microbiome and DAI in down‐regulation of RUNX2 and low PBM in female offspring induced by PPE.
**Figure S4.** Metabolic profiling of maternal and female fetal serum.
**Figure S5.** DAI could alleviate PPE‐induced *Hoxd12* low expression and PBM reduction.
**Figure S6.** *Hoxd12* mediated the low osteogenesis function and PBM in female offspring rats induced by PPE.
**Figure S7.** Promoted osteogenic differentiation in PPE‐BMSCs induced by DAI.
**Figure S8.** Upregulated *Hoxd12* in PPE‐BMSCs by DAI through ERβ/Kat6a.
**Figure S9.** Down‐regulation of *Hoxd12* in female offspring induced by PPE is not associated with estrogen, prednisone, and prednisolone in fetal serum.
**Figure S10.** Effect of maternal DAI supplementation on multiorgan's toxicology development.

**Table S1.** Effects of PPT on pregnancy outcome, and the development of fetus and bone.
**Table S2.** Population characteristics of maternal fecal specimens collected.
**Table S3.** Characteristics of peripheral serum from maternal and neonatal.
**Table S4.** Primers for real‐time quantitative PCR.

## Data Availability

The data that support the findings of this study are openly available in the NCBI Sequence Read Archive (SRA) at https://www.ncbi.nlm.nih.gov/bioproject/, reference numbers PRJNA1241687 (https://www.ncbi.nlm.nih.gov/sra/PRJNA1241687), PRJNA1243860 (https://www.ncbi.nlm.nih.gov/sra/PRJNA1243860), and PRJNA1241537 (https://www.ncbi.nlm.nih.gov/sra/PRJNA1241537). The analysis of RNA sequencing, 16s rRNA microbiome sequencing, and untargeted metabolomics was conducted by Novomagic (https://magic.novogene.com) and Metware Cloud (https://cloud.metware.cn). The data and scripts used are saved in GitHub (https://github.com/machihn/ChiMa-iMeta.git). Supplementary materials (figures, tables, graphical abstract, slides, videos, Chinese translated version, and update materials) may be found in the online DOI or iMeta Science http://www.imeta.science/.
