# Supplementary material for: Maternal gut microbiota‐derived daidzein prevents osteoporosis in female offspring following prenatal prednisone exposure
Source: Imeta. 2025 Apr 28;4(4):e70037. doi: 10.1002/imt2.70037 (PMC12371263; doi:10.1002/imt2.70037)
Supplement: Supplementary file 1 — Figure S1. Effects of PPT/PPE on maternal gut microbiome, male offspring long bone development, and PBM in male offspring. Figure S2. Effects of PPE on osteogenesis function in female offspring. Figure S3. Involvement of maternal gut microbiome and DAI in down‐regulation of RUNX2 and low PBM in female offspring induced by PPE. Figure S4. Metabolic profiling of maternal and female fetal serum. Figure S5. DAI could alleviate PPE‐induced Hoxd12 low expression and PBM reduction. Figure S6. Hoxd12 mediated the low osteogenesis function and PBM in female offspring rats induced by PPE. Figure S7. Promoted osteogenic differentiation in PPE‐BMSCs induced by DAI. Figure S8. Upregulated Hoxd12 in PPE‐BMSCs by DAI through ERβ/Kat6a. Figure S9. Down‐regulation of Hoxd12 in female offspring induced by PPE is not associated with estrogen, prednisone, and prednisolone in fetal serum. Figure S10. Effect of maternal DAI supplementation on multiorgan's toxicology development. [file IMT2-4-e70037-s001.docx]

**Supporting information to**

**Maternal gut microbiota-derived daidzein prevents osteoporosis in female offspring following prenatal prednisone exposure**

**Running title:** Daidzein prevents offspring susceptibility to fetal originated-osteoporosis

Chi Ma^1#^, Hangyuan He^1#^, Kunpeng Wang^5#^, Juanjuan Guo^3,4^, Liang Liu^1^, Yuting Chen^3^, Bin Li^1^, Hao Xiao^1,4^, Xufeng Li^1^, Xiaoqian Lu^2^, Tingting Wang^2^, Yinxian wen^1,4^, Hui Wang^2,4*^, Liaobin Chen^1,4*^

^1^Department of Orthopaedic Surgery, Division of Joint Surgery and Sports Medicine, Zhongnan Hospital of Wuhan University, Wuhan 430071, China;

^2^Department of Pharmacology, Basic Medical School of Wuhan University, Wuhan 430071, China;

^3^Department of Obstetrics and Gynaecology, Zhongnan Hospital of Wuhan University, Wuhan 430071, China;

^4^Hubei Provincial Key Laboratory of Developmentally Originated Diseases, Wuhan 430071, China;

^5^Department of Obstetrics and Gynaecology, Wuhan Hospital of Traditional Chinese Medicine, Wuhan 430071, China.

^#^These authors contributed equally: Chi Ma, Hangyuan He, Kunpeng Wang

^*^Correspondence: [lbchen@whu.edu.cn](mailto:lbchen@whu.edu.cn) (Liaobin Chen); [wanghui19@whu.edu.cn](mailto:wanghui19@whu.edu.cn) (Hui Wang)

**
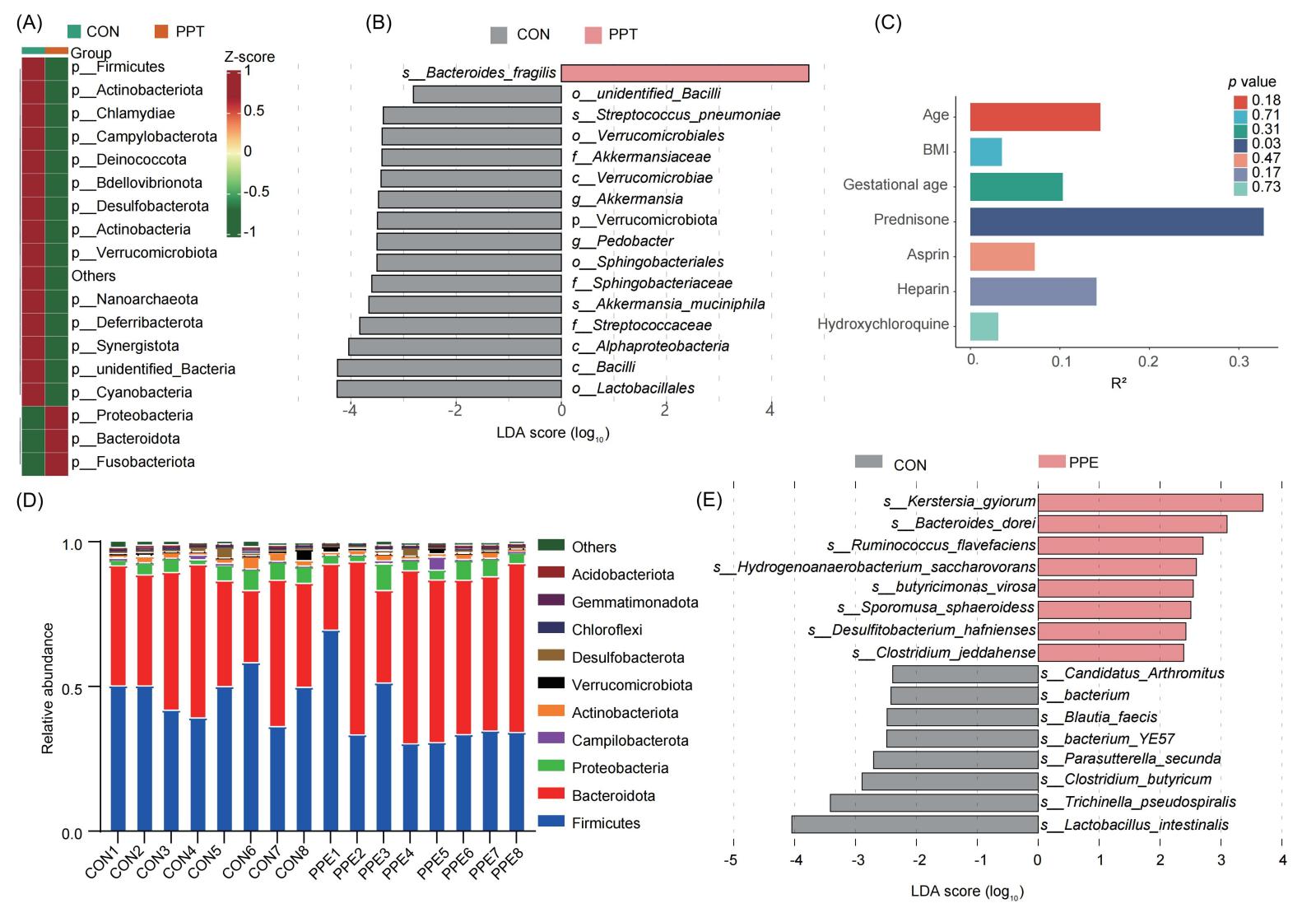
Figure S1 Effects of PPT/PPE on maternal gut microbiome, male offspring long bone development and PBM in male offspring.** (A) heatmap of relative abundance at the phylum level of human maternal gut microbiota. (B) LEfSe analysis (LDA > 2) in human maternal gut microbiota. (C) the CCA plot showing the relationship of factors and the maternal gut microbiota variation. (D) top 10 in terms of relative abundance at the phylum level in maternal gut microbiota. (E) LEfSe analysis (LDA > 2) in maternal gut microbiota. *n* = 8 for 16s rRNA sequencing of maternal gut microbiota. *n* = 14 in CON and *n* = 11 in PPT for 16s rRNA sequencing of human maternal gut microbiota. Mean ± SEM, *n* = 14 in CON and *n* = 11 in PPT for 16s rRNA sequencing of human maternal gut microbiota, *n* = 8 for 16s rRNA sequencing of rat maternal gut microbiota. ^*^*p* < 0.05, ^**^*p* < 0.01 *vs.* CON. PPT, prenatal prednisone therapy; PPE, prenatal prednisone exposure; LEfSe, linear discriminant analysis of effect size; LDA, linear discriminant analysis score; CON, control.

**
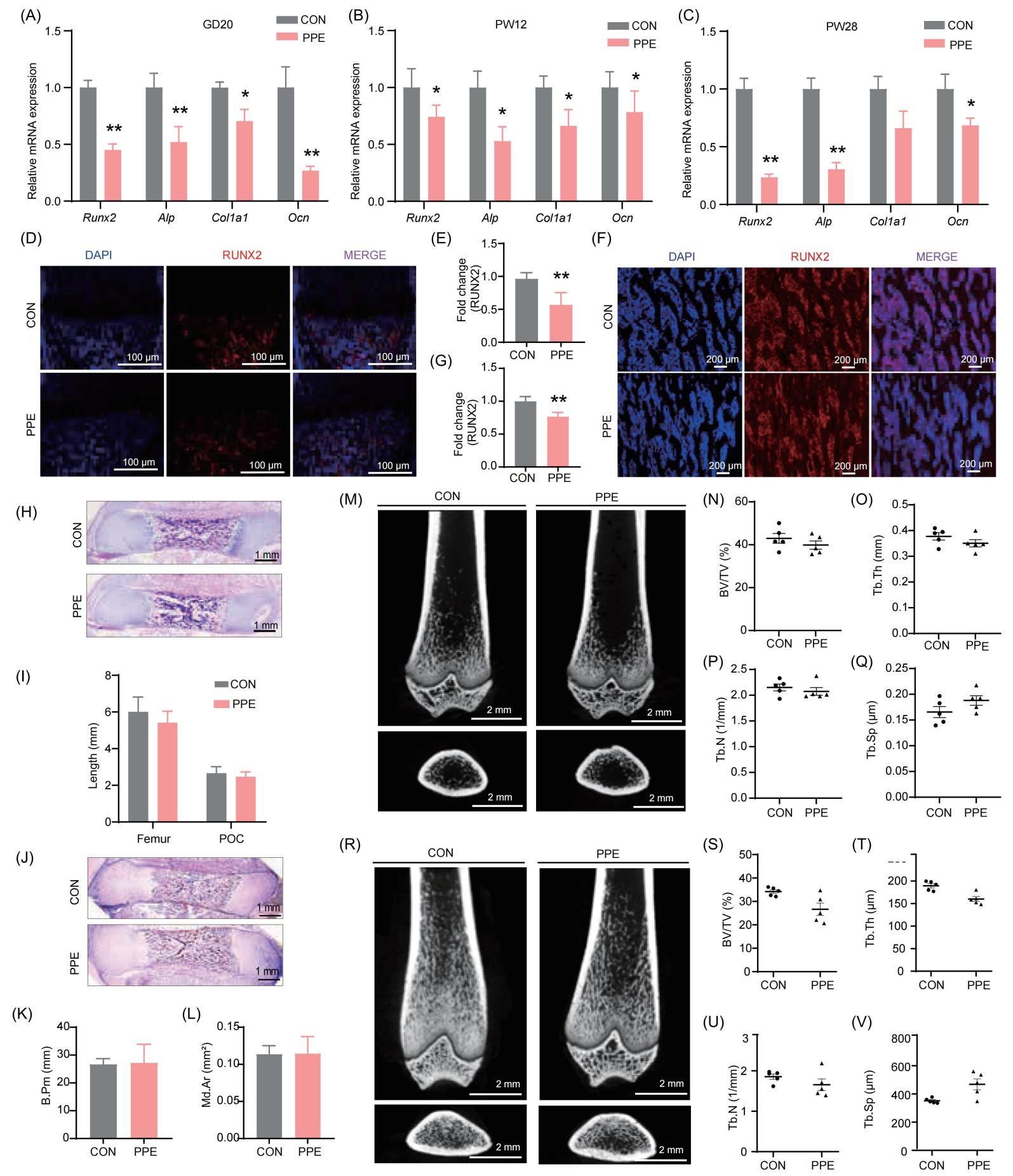
**

**Figure S2 Effects of PPE on osteogenesis function in female offspring.** (A-C) osteogenesis function-related marker genes by RT-qPCR. (D and E) representative images of immunofluorescence staining and semi-quantification analysis of RUNX2 in GD20 female offspring femur, scale bar = 200 μm. (F and G) representative images of immunofluorescence staining and semi-quantification analysis of RUNX2 in PW 12 female offspring femur, scale bar = 200 μm. (H) femur H&E staining in male fetal. (I) quantitative analysis of total femur and POC length for H&E staining in male rat. (J) femur Vonkossa staining in male fetal rats. (K and L) quantitative analysis of B. Pm and Md. Ar for Vonkossa staining in the male rat. (M-Q) representative images and quantitative analysis of micro-CT in PW12 male offspring, scale bar = 2 mm. (R-V) representative images and quantitative analysis of micro-CT in PW28 male offspring, scale bar = 2 mm. Mean ± SEM, *n* = 8 for RT-qPCR, *n* = 3 for immunofluorescence staining, H&E and Vonkossa staining, *n* = 5 for micro-CT. ^*^*p* < 0.05, ^**^*p* < 0.01 *vs.* CON. RT-qPCR, real-time quantitative polymerase chain reaction; RUNX2, Runt-related transcription factor 2; H&E, hematoxylin, and eosin; POC, primary ossification center; B. Pm, bone trabecula perimeter; Md. Ar, mineralized area; CT, computed tomography; PW, postnatal week; *Alp*, alkaline phosphatase; *Col1a1*, collagen type I Alpha 1; *Ocn*, Osteocalcin; BV/TV, bone volume/tissue volume; Tb. N, trabecular number; Tb. Th, trabecular thickness; Tb. Sp, trabecular separation.

**
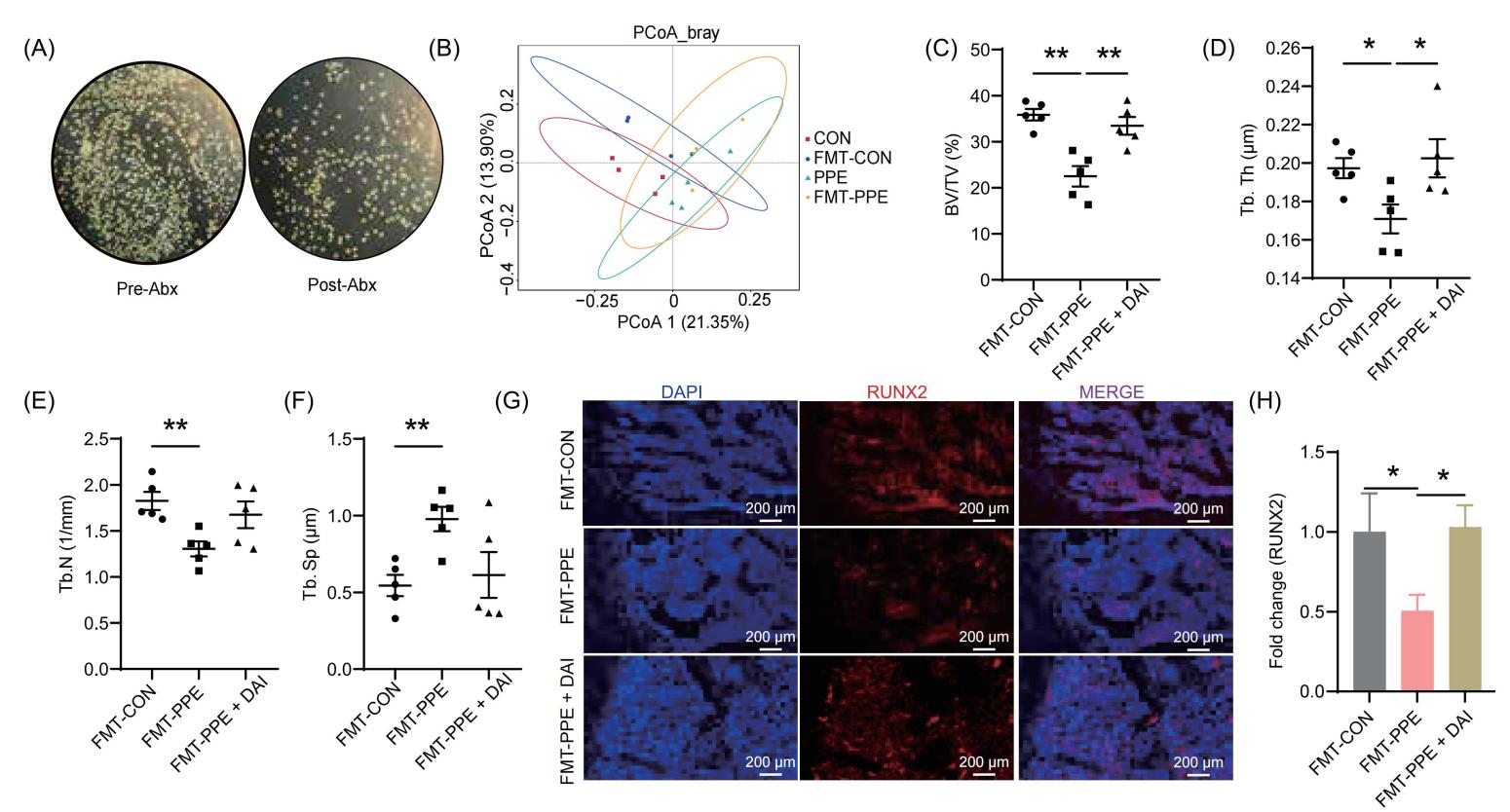
**

**Figure S3 Involvement of maternal gut microbiome and DAI in down-regulation of RUNX2 and low PBM in female offspring induced by PPE.** (A) representative image of the fecal colony from the pre-Abx and post-Abx rat. (B) PCoA plot analysis of GD20 maternal gut microbiota. (C-F) quantitative analysis of micro-CT. (G and H) representative immunofluorescence images and semi-quantification analysis of RUNX2, scale bar = 200 μm. Mean ± SEM, *n* = 3 for fecal colony; *n* = 4 for 16s rRNA sequencing; *n* = 5 for micro-CT, *n* = 3 for immunofluorescence. ^*^*p* < 0.05, ^**^*p* < 0.01 *vs.* corresponding control. DAI, daidzein; PBM, peak bone mass; PPE, prenatal prednisone exposure; pre-Abx, before antibiotic treatment; post-Abx, post antibiotic treatment; PCoA, principal co-ordinates analysis; GD, gestational day; FMT-CON, pregnant rats transplanted with fecal microbiota from control maternal group; FMT-PPE, pregnant rats transplanted with fecal microbiota from PPE maternal group; FMT-PPE + DAI, pregnant rats transplanted with fecal microbiota from PPE maternal group and supplemented with DAI.

**
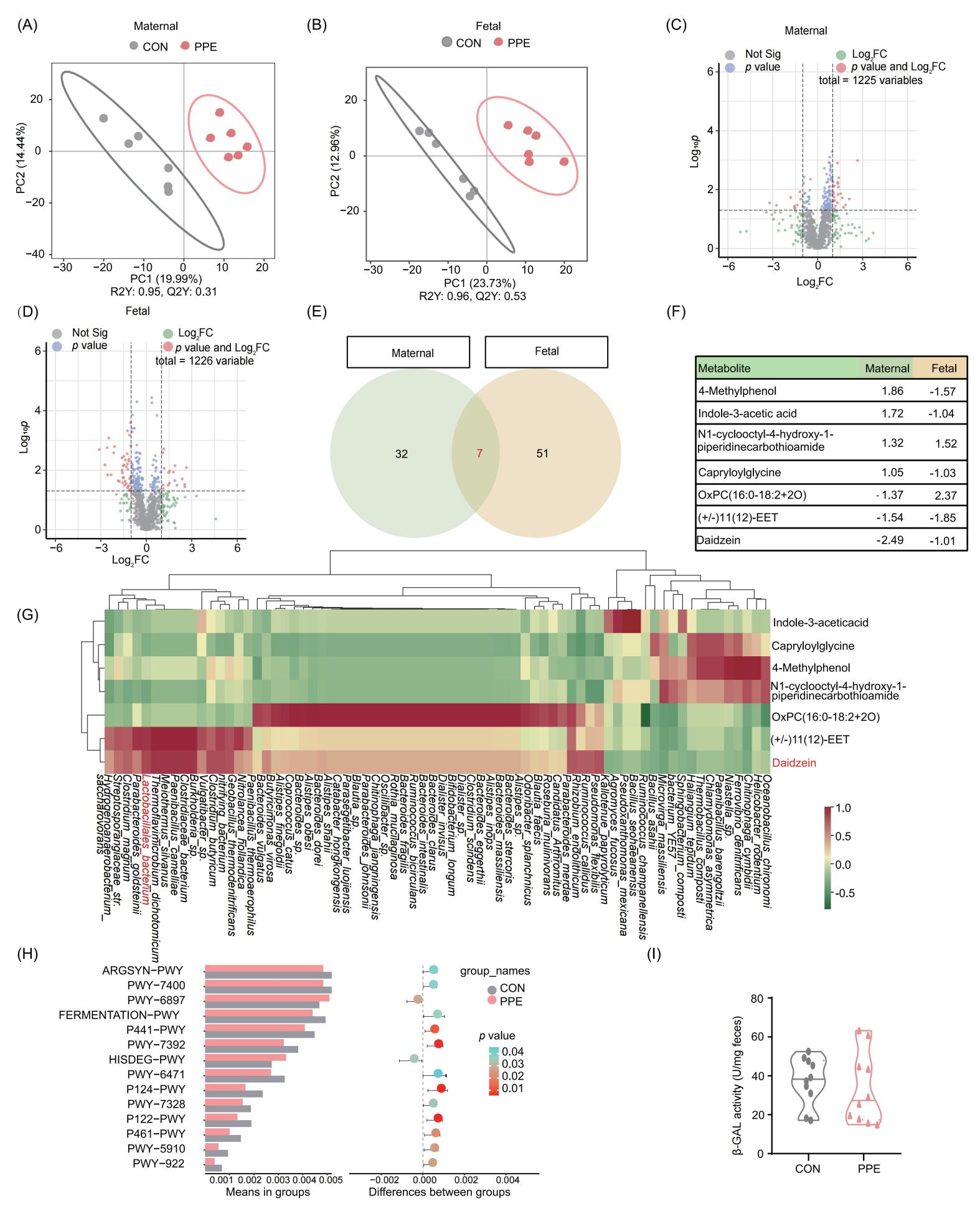
**

**Figure S4 Metabolic profiling of maternal and female fetal serum.** (A and B) PCA analysis of metabolic profile of maternal and female fetal serum. (C and D) volcano plot of untargeted metabolic profile for rat maternal and fetal serum. (E) the shared differentiated metabolites in rat maternal and fetal serum. (F) fold change (Log_2_) of the shared differentiated metabolites in rat maternal and fetal serum. (G) correlation analysis of differential gut microbiota in maternal rats with the concentration of seven shared metabolisms in fetal serum. (H) predicated microbiome functions by PICRUSt. (I) β-GAL activity in maternal feces shown as violin plots. Mean ± SEM, *n* = 6 for untargeted metabolic profiling. ^*^*p* < 0.05, ^**^*p* < 0.01 *vs.* corresponding control. PCA, principal component analysis; OxPC, oxidized phosphatidylcholines; EET, epoxyeicosatrienoic acids; PICRUSt, phylogenetic investigation of communities by reconstruction of unobserved states; β-GAL, β-galactosidase; ARGSYN-PWY: L-arginine biosynthesis I; PWY-7400: L-arginine biosynthesis IV; PWY-6897: thiamine salvage II; FERMENTATION-PWY: mixed acid fermentation; P441-PWY: superpathway of N-acetylneuraminate degradation; PWY-7392: taxadiene biosynthesis; PWY-6471: peptidoglycan biosynthesis IV; P124-PWY: Bifidobacterium shunt; PWY-7328:superpathway of UDP-glucose-derived O-antigen building blocks biosynthesis; P122-PWY: heterolactic fermentation; P461-PWY: hexitol fermentation to lactate, formate, ethanol and acetate; PWY-5910: superpathway of geranylgeranyldiphosphate biosynthesis I; PWY-922: mevalonate pathway I.

**
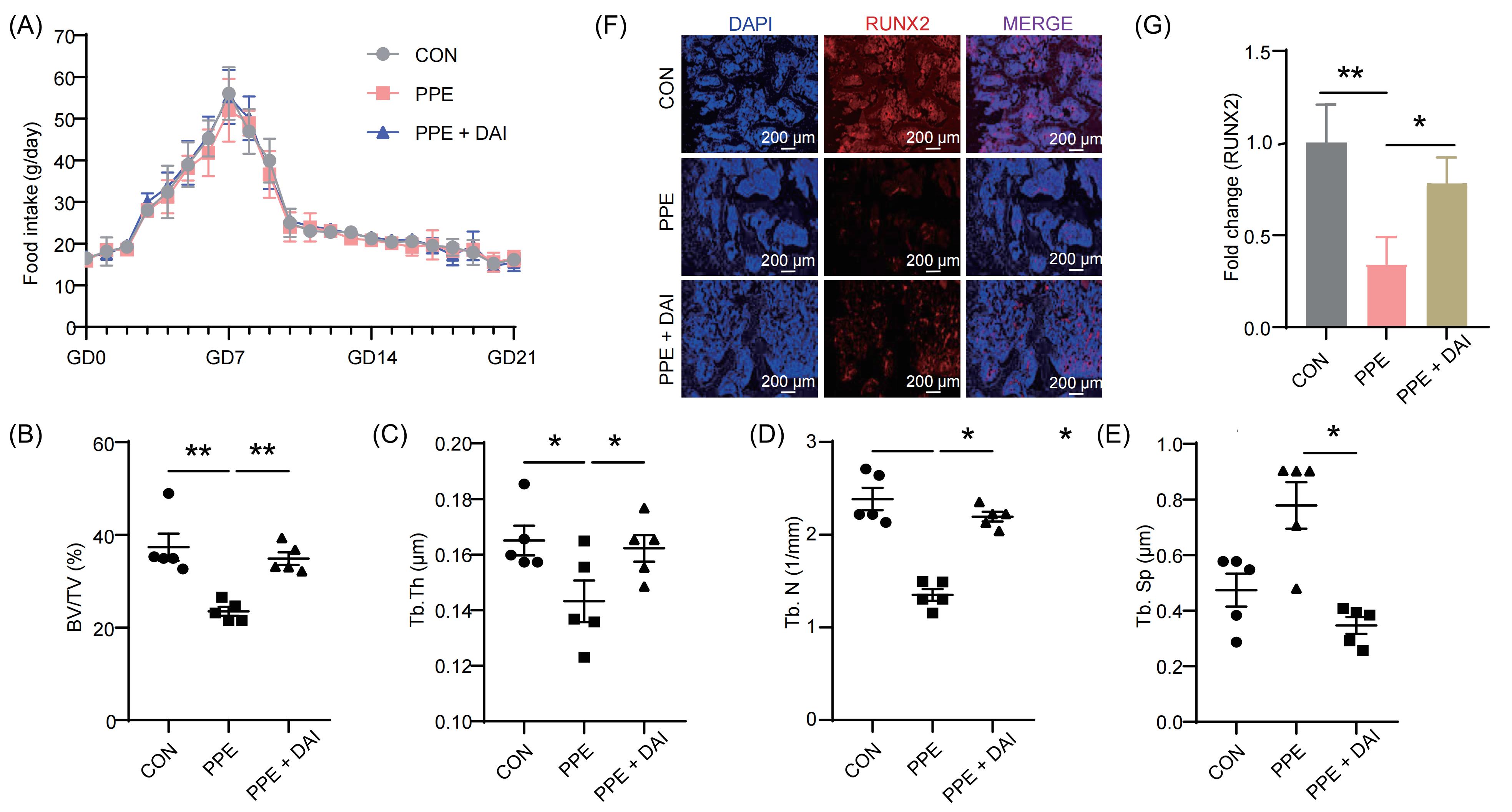
**

**Figure S5** **DAI could alleviate PPE-induced *Hoxd12* low expression and PBM reduction.** (A) maternal food intake; (B-E) quantitative analysis of micro-CT. (F and G) representative immunofluorescence images and semi-quantification analysis of RUNX2, scale bar = 200 μm. Mean ± SEM, *n* = 5 for micro-CT, *n* = 3 for immunofluorescence staining. ^*^*p* < 0.05, ^**^*p* < 0.01 *vs.* corresponding control. *Hoxd12*, homeobox D12.

**
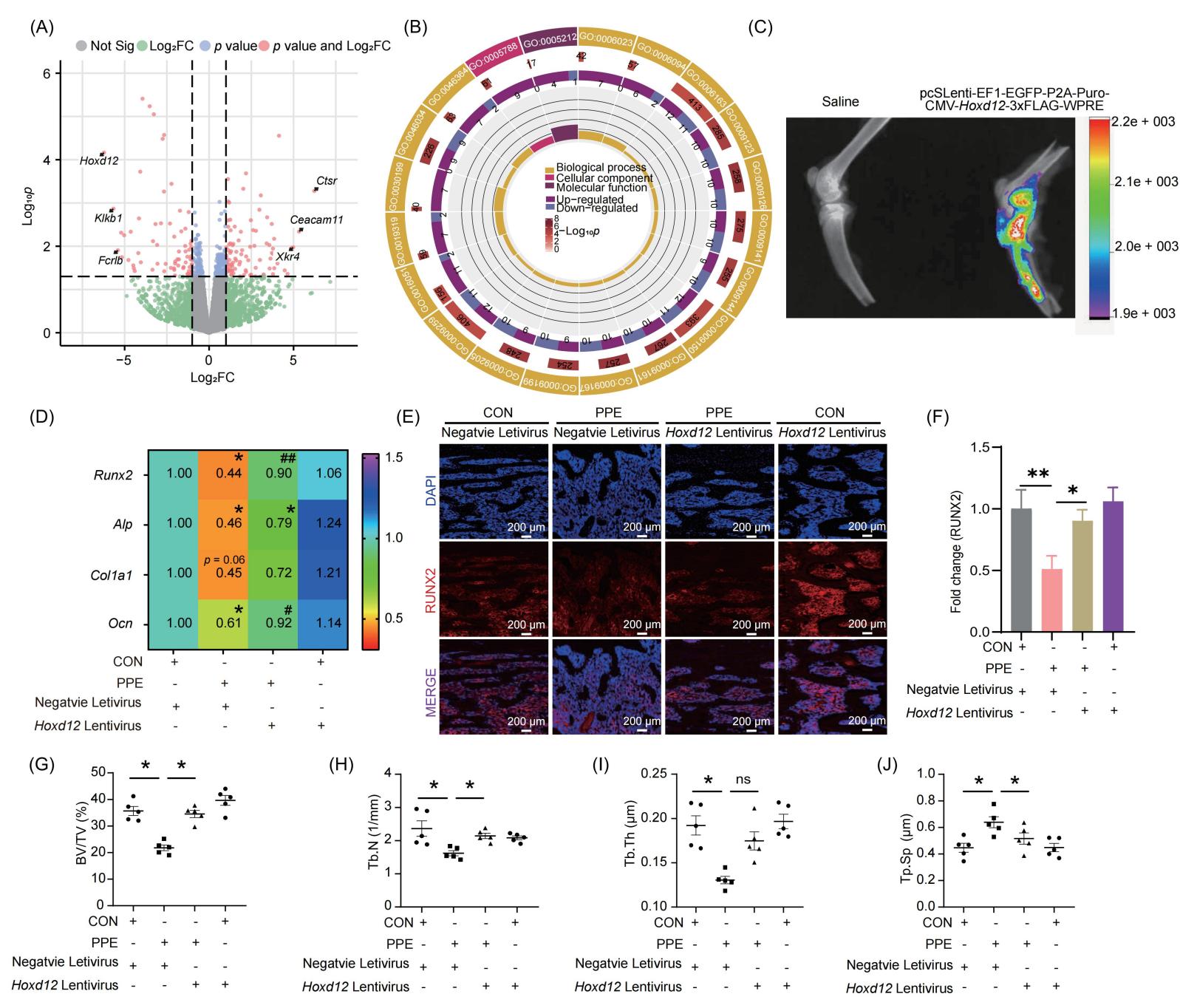
**

**Figure S6 *Hoxd12* mediated the low osteogenesis function and PBM in female offspring rats induced by PPE.** (A) volcano plot of RNA-seq. (B) GO analysis of RNA-seq. (C) eGFP fluorescence expression. (D) heatmap for osteogenesis differentiation-related marker genes. (E and F) representative immunofluorescence images and semi-quantification analysis of RUNX2, scale bar = 200 μm. (G-J) quantitative analysis of micro-CT. (M and N) quantitative analysis of HOXD12. Mean ± SEM, *n* = 8 for RT-qPCR, *n* = 3 for immunofluorescence and immunohistochemistry staining, *n* = 5 for micro-CT. ^*^*p* < 0.05, ^**^*p* < 0.01 *vs.* corresponding control. *Hoxd12*, homeobox D12; PBM, peak bone mass; PPE, prenatal prednisone exposure; RNA-seq, RNA sequence; GO, gene ontology; PW, postnatal week; eGFP, enhanced green fluorescent protein.

**
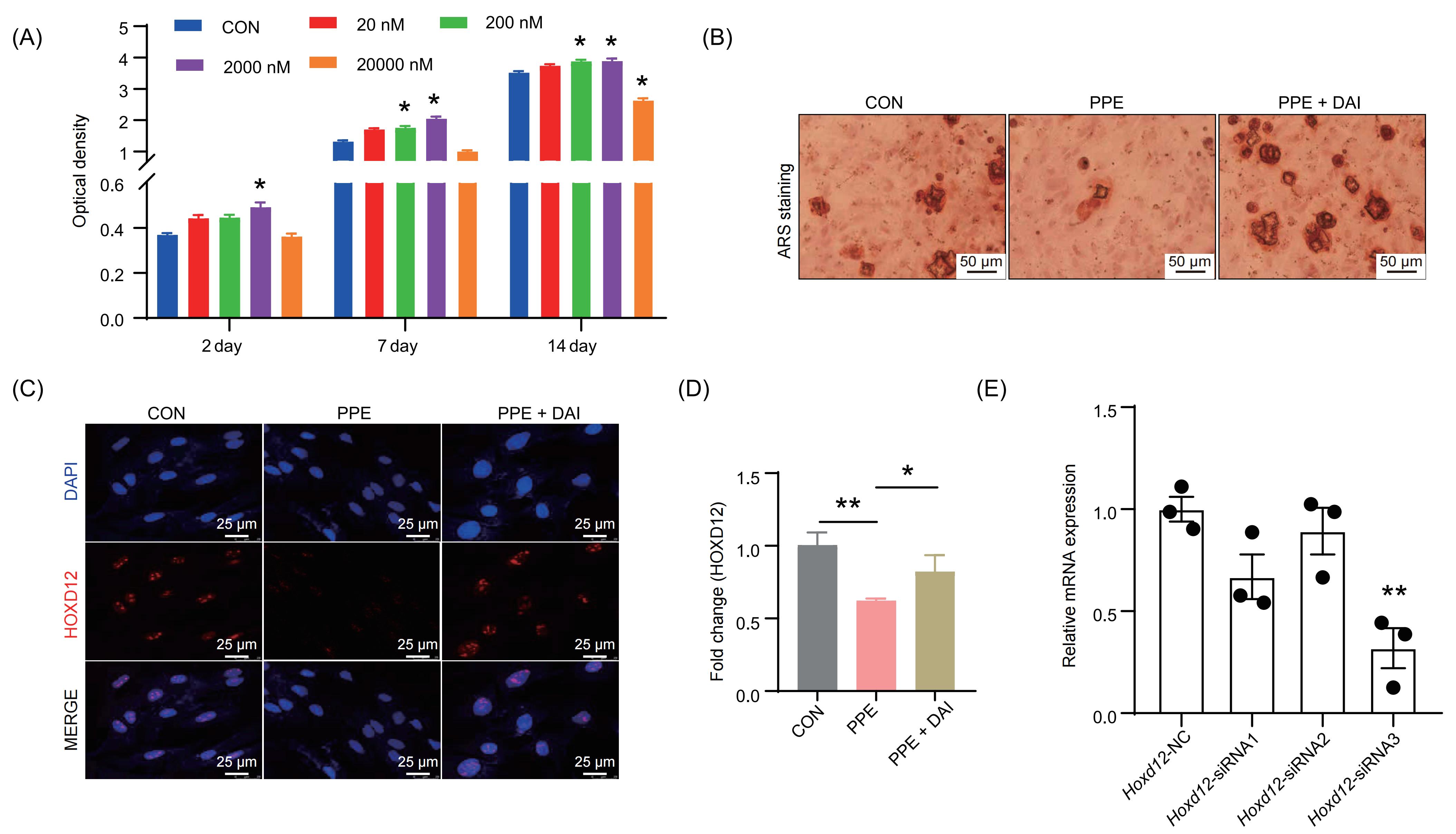
**

**Figure S7 Promoted osteogenic differentiation in PPE-BMSCs induced by DAI.** (A) BMSCs activity treated with different concentrations or time of DAI by CCK-8. (B) ARS staining. (C and D) representative immunofluorescence images and semi-quantitative analysis of HOXD12, scale bar = 25 μm. (E) Effect of siRNA on *Hoxd12* in BMSCs. Mean ± SEM, *n* = 6 for CCK8, *n* = 3 for ARS staining and immunofluorescence, *n* = 3 for RT-qPCR. ^*^*p* < 0.05, ^**^*p* < 0.01 *vs.* corresponding control. BMSCs, bone marrow mesenchymal stem cells; PPE-BMSCs, primary BMSCs in offspring femur from PPE group; CCK8, Cell Counting Kit-8; ARS, Alizarin Red S; siRNA, Small interfering RNA; CON-BMSCs, primary BMSCs in offspring femur from control group; NC, negative control.

**
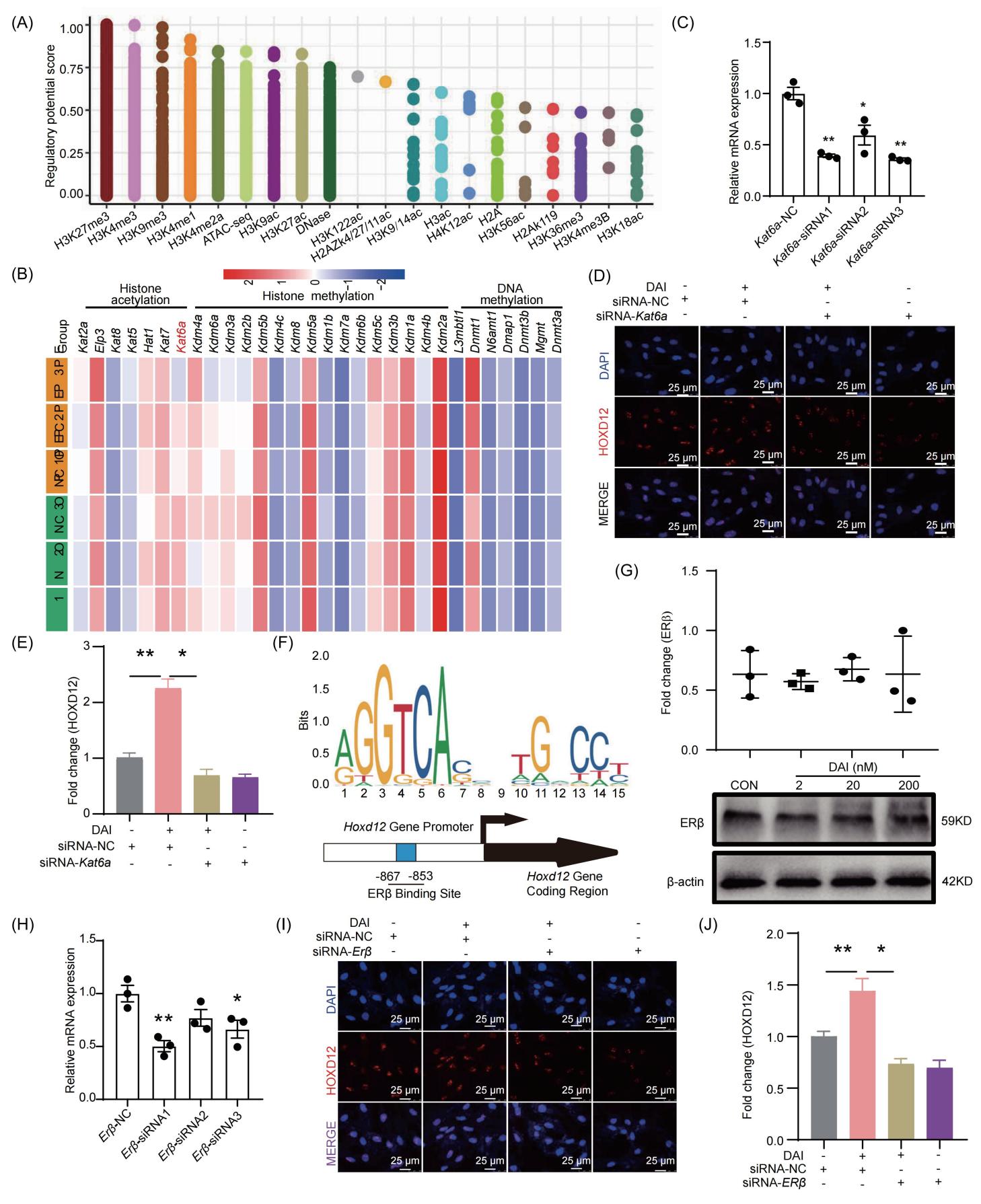
**

**Figure S8 Upregulated *Hoxd12* in PPE-BMSCs by DAI through ERβ/*Kat6a*.** (A) common epigenetic modifications of *Hoxd12* identified by Cistrome Data Browser. (B) heatmap of key epigenetic modifiers based on RNA-seq analysis from GD20 female fetal femur. (C) Effect of siRNA on *Kat6a* in BMSCs. (D and E) representative immunofluorescence images and semi-quantitative analysis of HOXD12, scale bar = 25 μm. (F) the promoter region ERβ binding site are numbered relative to the transcription start site. (G) representative immunoblots and quantification analysis of ERβ expression. (H) Effect of siRNA on *Erβ* in BMSCs. (I and J) representative immunofluorescence images and semi-quantitative analysis of HOXD12, scale bar = 25 μm. Mean ± SEM, *n* = 3 for RT-qPCR, Western blot and immunofluorescence. ^*^*p* < 0.05, ^**^*p* < 0.01 *vs.* corresponding control. ERβ, estrogen receptor β; *Kat6a*, lysine acetyltransferase 6A; RNA-seq, RNA sequence.


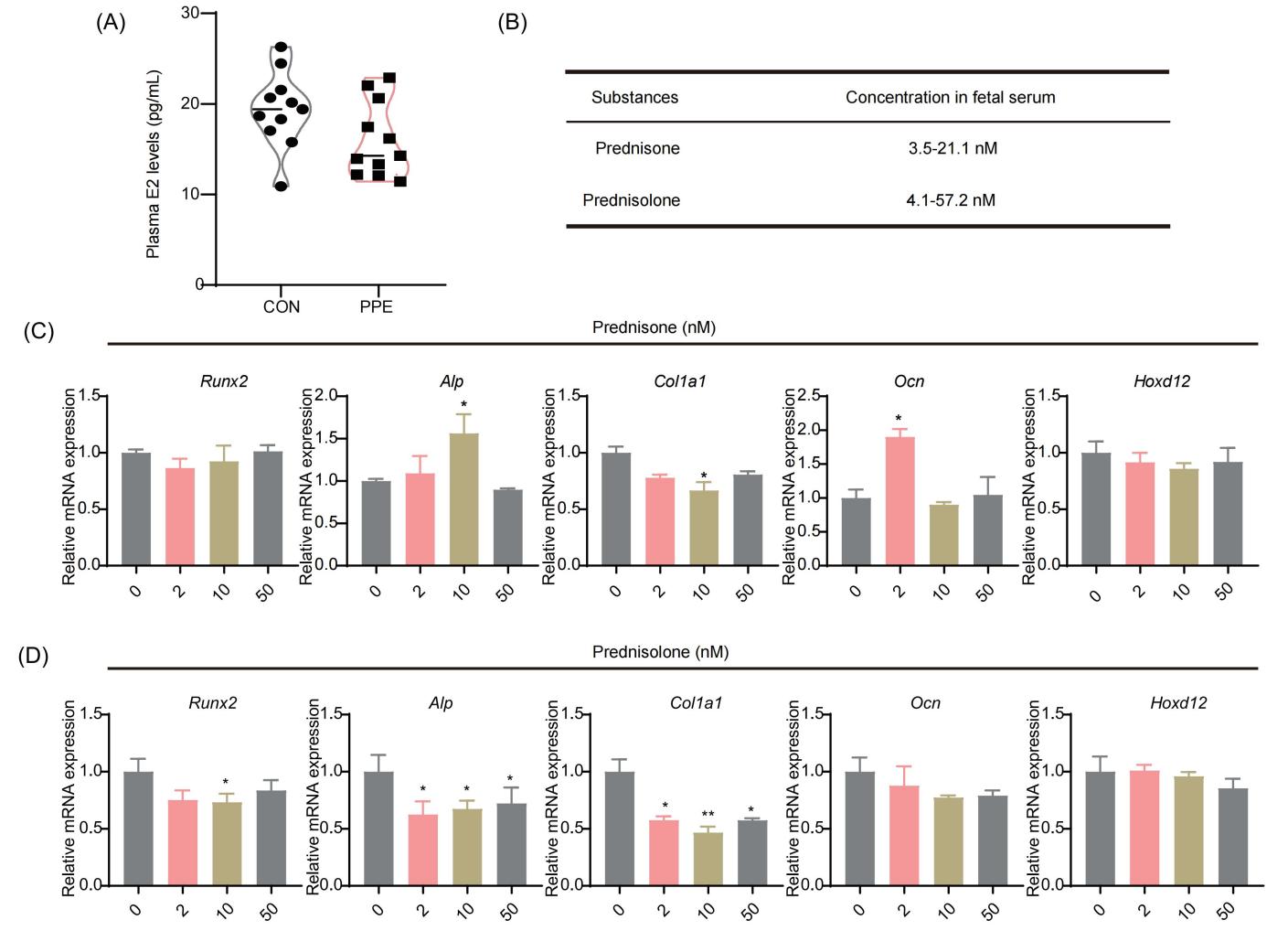


**Figure S9** **Down-regulation of *Hoxd12* in female offspring induced by PPE is not associated with estrogen, prednisone, and prednisolone in fetal serum.** (A) the E2 level in female fetal serum. (B) the prednisone and prednisolone level in female fetal serum by LC-MS/MS. (C and D) the expression of osteogenesis differentiation-related marker genes and *Hoxd12*. Data in (A) are presented as the median ± interquartile range, and data in other figures are presented as the Mean ± SEM, *n* = 11 for ELISA, *n* = 6 for LC-MS/MS, *n* = 6 for RT-qPCR. ^*^*p* < 0.05, ^**^*p* < 0.01 *vs.* corresponding control. E2, estradiol; LC-MS/MS, liquid chromatography/tandem mass spectrometer.

**
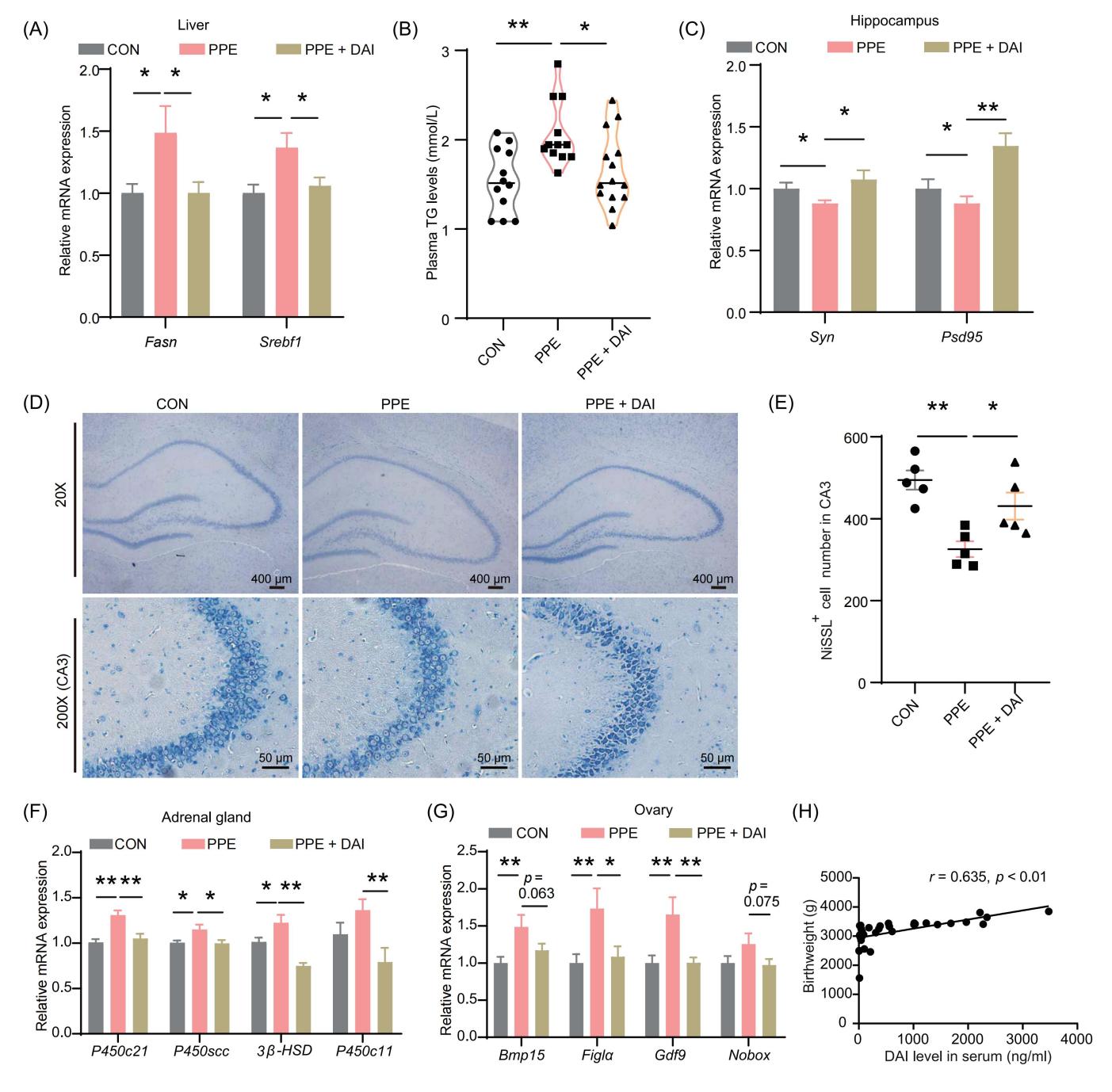
**

**Figure S10 Effect of maternal DAI supplementation on multiorgan’s toxicology development.** (A) hepatic lipid synthesis genes. (B) plasma TG level shown as violin plots. (C) hippocampal neurodevelopment-related marker genes. (D and E) Nissl staining of hippocampus and quantification of Nissl^+^ cell number in CA3 (*n* = 5), scale bar = 400 μm or 50 μm. (F) adrenal steroid synthesis marker genes. (G) ovarian follicular development marker genes. (H) correlation analysis between daidzein levels and birth weight in clinical female infants. Data in (B) are presented as the median ± interquartile range, and data in other figures are presented as the Mean ± SEM, *n* = 8-12 for RT-qPCR, *n* = 3 for Nissl staining, ^*^*p* < 0.05, ^**^*p* < 0.01 *vs.* corresponding control. TG, triglyceride; Fasn, fatty acid synthase; *Srebf1*, sterol regulatory element binding transcription factor 1;*Syn*, synapsin; *Psd95*, postsynaptic density 95; *P450c11*, cytochrome P450 family 11; *P450c21*, cytochrome P450 family 21; *P450scc*, cytochrome P450 family 11 subfamily A member 1; *3β-HSD*, hydroxy-delta-5-steroid dehydrogenase; *Bmp15*, bone morphogenetic protein 15; *Figl*α, folliculogenesis specific BHLH transcription factor; *Gdf9*, growth differentiation factor 9; *Nobox*, NOBOX oogenesis homeobox.
